# Using Real Time Measurements to Derive the Indoor and Outdoor Contributions of Submicron Particulate Species and Trace Gases

**DOI:** 10.3390/toxics10040161

**Published:** 2022-03-29

**Authors:** Evdokia Stratigou, Sébastien Dusanter, Joel Brito, Emmanuel Tison, Véronique Riffault

**Affiliations:** Centre for Energy and Environment, IMT Nord Europe, University of Lille, Institut Mines-Télécom, F-59000 Lille, France; sebastien.dusanter@imt-nord-europe.fr (S.D.); joel.brito@imt-nord-europe.fr (J.B.); emmanuel.tison@imt-nord-europe.fr (E.T.)

**Keywords:** indoor air quality, indoor air chemistry, particulate matter (PM), volatile organic compounds (VOC), apportionment methodology

## Abstract

The indoor environment is usually more polluted than outdoors due to emissions of gas and particle-phase pollutants from multiple sources, leading to their accumulation on top of the infiltration of outdoor pollution. While it is widely recognized that negative health effects arise from the exposure to outdoor air pollution, exposure to indoor pollutants also needs to be well assessed since we spend most of our time (~90%) breathing indoors. Indoor concentrations of pollutants are driven by physicochemical processes and chemical transformations taking place indoors, acting as sources and/or sinks. While these basic concepts are understood, assessing the contribution of each process is still challenging. In this study, we deployed online instrumentation in an unoccupied room to test a methodology for the apportionment of indoor and outdoor pollutant sources. This method was successfully applied to the apportionment of PM_1_ and VOCs, however, there are limitations for reactive gases such as O_3_. The results showed that this unoccupied indoor environment acts as a source of VOCs and contributes 87% on OVOCs and 6% on C_x_H_y_, while it acts as a sink for particles, likely due to losses through volatilization up to 60%.

## 1. Introduction

The indoor environment is usually more polluted than just the infiltrated ambient air due to the presence of additional sources emitting pollutants such as volatile organic compounds (VOCs, a glossary can be found in the Supplementary Information) and particulate matter (PM) in a reduced volume [1]. Nowadays, as part of climate change mitigation measures, improving energy efficiency in the built environment leads to an increasing demand on reducing air exchange with outdoors, which in turn increases human exposure to indoor pollution [2]. While it has been recognized worldwide that negative health effects correlate with outdoor pollutant levels, we spend most of our time breathing indoors. In particular, in industrially developed countries, it is estimated that people spend on average 90–95% of their time inside buildings and an additional 5.5% inside vehicles [2,3]. According to the World Health Organization (WHO) [4], 3.8 million premature deaths worldwide were attributable to household air pollution in 2016, accounting for 7.7% of the global mortality. Thus, a good understanding of both the sources and the sinks of indoor pollutants is important to design strategies aiming at reducing personal exposure to harmful species.

Due to their numerous health impacts on the respiratory and cardiovascular systems, as well as their genotoxicity and carcinogenicity [5,6,7,8], several countries have established air quality standards on ambient mass concentrations of PM with an aerodynamic diameter lower than 10 µm (PM_10_, termed inhalable PM) or 2.5 µm (PM_2.5_, termed fine PM). PM of even smaller sizes (e.g., PM_1_ with a diameter lower than 1 µm) are of particular interest since they can deposit more efficiently in the lower respiratory tract and access the circulatory system, also impacting other organs [9,10]. Furthermore, smaller particles are typically observed at higher number concentrations and therefore exhibit an overall larger surface area, which can act as a medium for the adsorption of organic pollutant [11]. However, no official regulations exist nowadays for this fraction. Only a few studies have assessed the specific impact of PM_1_ on human health [12,13,14,15,16,17]. For instance, it has been found that a daily increase of 10 μg m^−3^ is significantly associated with a 0.6% increase in total mortality [12] as well as an increased risk of emergency hospital visits [13] or with at least one lower respiratory symptom [15,17]. On the other hand, a wide range of VOCs can cause allergies, mucous membrane irritation and/or inflammation, systemic effects, such as fatigue and difficulty in concentrating, respiratory problems such as asthma and wheezing, as well as toxic effects such as carcinogenicity [18,19,20,21,22]. Official regulations for ambient VOCs (outdoors) concerns only benzene [23]. However, guide values have been established in 2011 by the French law for formaldehyde and benzene for establishments open to the public [24]. Besides, the French Agency for Food, Environmental and Occupational Health & Safety (ANSES) also recommends guide values for indoor air concerning ten additional gaseous pollutants taking into account short- and long-term exposures [25]. Indoor concentrations of particles and gases are driven by complex physical and chemical processes acting as sources—of primary (direct emission) or secondary (products of chemical transformations) nature, and sinks. Primary pollutants include species originating either from outdoor due to air exchange or from indoors (emissions from building materials, consumer products, occupants’ activities), while sinks can be attributed to air exchange and adsorption/deposition processes for gases/particles, respectively. Secondary pollutants originate from gas-to-particle conversion processes occurring in the gaseous phase or at the gas-surface interface. The extent to which specific pollutants observed indoors are the result of outdoor infiltration or indoor processes mentioned above is still difficult to quantify. Methodologies developed for source apportionment of indoor pollution are mainly based on complex source receptor or personal exposure modelling (i.e., Positive Matrix Factorization, chemical mass balance model, gas phase chemistry or kinetic process modelling) [26,27,28,29,30,31,32,33,34,35], which often require numerous assumptions (e.g., that highly correlated compounds come from the same source) [36] and/or sets of observations exhibiting a large temporal variability to be able to deconvolve the different sources.

The objective of this work was to assess a simpler methodology capable of apportioning indoor and outdoor contributions to the observed indoor concentrations of pollutants, including organic and inorganic particles and gases. The study was conducted in an unoccupied low energy building, where we combined high (time and mass) resolution measurements of PM_1_ and trace gases. In the following, we present indoor and outdoor contributions derived from the proposed methodology for the pool of pollutants mentioned above, discuss physicochemical processes impacting the composition of ambient infiltrated air, and address limitations associated to this methodology.

## 2. Materials and Methods

### 2.1. Measurement Facility

The measurement facility is located at the Institute Mines Telecom Nord Europe on the Douai Campus, Northern France. This building is mainly made of wood with a footprint of 12 m^2^ and a height of 2.4 m, leading to a surface to volume ratio of 0.4 m^2^/m^3^ (Supplementary Information, Figure S1). It is a so-called energy-efficient building with the French BBC label (Bâtiment Basse Consommation—France) [37]. The test room chosen for our study is equipped with a dual-flow ventilation system with heat recovery (KWL EC 60 Pro, HELIOS) providing an air flow rate of 17 m^3^ h^−1^. This system is equipped with an aluminum plate heat exchanger (efficiency higher than 70%), two fans with energy-saving EC motors for homogeneous air exchange and two efficient air filters (class G4). Ventilation was kept on during the whole measurement period. The room was kept unfurnished and unoccupied during the experiments to minimize resuspension and material sources, as well as direct emissions by human activities (cooking, cleaning, occupancy, etc.).

The spatial homogeneity of PM within the room was investigated using the 2 AEROTRACK instruments setup at various locations and heights, showing relative differences lower than 12% for all measured size ranges (0.3–0.5, 0.5–1.0, 1.0–3.0, 3.0–5.0, 5.0–10.0 μm), which is comparable to the instrumental uncertainty. The air exchange rate (α) was measured according to the ASTM E741-00 method [38] using CO_2_ (Air Liquide) as a gas tracer and found constant over time at a value of 0.54 ± 0.05 h^−1^ (*n* = 9). Particle penetration (P) was considered to occur only through the ventilation system since the building is tightly sealed to meet low energy requirements and was found to be 0.75 ± 0.09 for the PM_1_ fraction (Figure S2). Penetration factors for gases were not measured but were inferred from the analysis shown hereafter. More details can be found in a previous study from our group [39].

### 2.2. Sampling Setup, Instrumentation and Measurement Procedures

An intensive campaign took place from 7 to 19 December 2017 and included online measurements of chemically-resolved indoor and outdoor particle concentrations and trace gases. More specifically, PM measurements were sequentially performed indoors and outdoors using a Scanning Mobility Particle Sizer (SMPS, TSI model 3788, 10.2–414.2 nm), two optical particle counters (OPC, AEROTRAK 8220, five size bins from 0.3 to 5.0 μm) and an HR-ToF-AMS (Aerodyne Research Inc., AMS hereafter, chemically-resolved non-refractory PM_1_). In addition, trace gases were measured using a PTR-QiToFMS (Ionicon Analytik GmbH, Innsbruck, Austria, PTRMS hereafter) for VOCs, an NO_2_ CAPS (Aerodyne Research Inc., Billerica, MA, United States) for NO_2_ and an O342 analyzer (Environnement SA) for ozone. Table 1 summarizes the instrumentation used during the intensive campaign together with time resolution and limits of detection (LOD).

**Figure 1:**
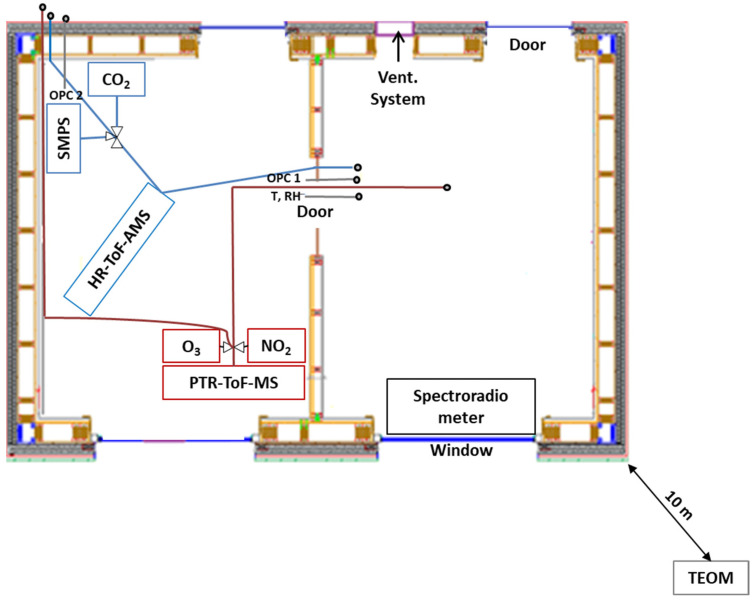
Schematic of the instrumentation room (**left**) and the experimental room (**right**).

Sequential indoor and outdoor particle concentration measurements were conducted continuously using two inlets made of stainless-steel and a valve-switching device that alternated the sampling between outdoor and indoor air every 5 min, resulting in a measurement every 10 min for each environment; the sampling line was shared between the AMS, the SMPS and the CO_2_ analyzer as shown in Figure 1 (blue line). A Nafion dryer was used to minimize moisture upstream the AMS and SMPS. The AMS measured the non-refractory PM_1_ (NR-PM_1_) [40,41] with a time resolution of 5 min including a 50-s flush of the inlet before acquiring data. Measurements were performed using 5 cycles of 10 s in MS mode (V-mode) and 20 s in PToF mode. Due to issues with ion optics, the W-mode data were not used. Blank measurements were performed each day for 10 min by guiding the ambient air through a HEPA particle filter. Figure S3, panel b in SI depicts the detailed setup. 

Calibrations of the inlet flow, the particle size and NO_3_ ionization efficiency (IE), as well as the relative ionization efficiencies (RIE) of nitrate, sulfate and ammonium for the AMS measurements were performed twice during the campaign, using pure ammonium nitrate (NH_4_NO_3_) and ammonium sulfate ((NH_4_)_2_SO_4_) particles and the setup shown in Figure S3, panel a, following the procedures recommended by the manufacturer (e.g., [42]). 

During this campaign, PTRMS measurements [43,44] were automatically switched between indoor and outdoor every 5 min using solenoid valves (SV). The zeros were performed automatically every 70 min for a duration of 20 min (a total of 90-min cycle). The measurement time resolution was set to 10 s for each environment including zeroing. Both the O_3_ and NO_2_ analyzers were connected to the same sampling system with the same measurement sequence and time resolution (Figure 1, red line). For this study, the PTRMS reactor was operated with a voltage of 960 V, a temperature of 70 °C, and a pressure of 3.8 mbar resulting in an electric field (E/N) of 137 Td. The two 15-m long sampling lines were made of ¼” PFA tubing. A PFA filter, changed every week, was used at the entrance of the line to filter aerosols. Ambient air was sampled at a total flow rate of 10 sLPM, leading to a residence time lower than 2 s in the sampling line. Calibrations have been conducted before (*n* = 6), during (*n* = 1) and after the end of the intensive campaign (*n* = 1). Section 1 in SI describes in detail the calibration procedure.

### 2.3. Data Treatment for Online Mass Spectrometers

The AMS dataset was analyzed using Squirrel v.1.60 and Pika v.1.20 module in the Igor Pro version 6.37 (Wavemetrics Inc.) software. 

Nitrate measured by AMS comes from NO^+^ and NO_2_^+^ fragments. These fragments can originate from organic molecules such as RONO2 or inorganic molecules such as NH_4_NO_3_. According to Farmer et al., 2010 [45], the measured nitrate can be separated into these two types by applying the following equations. This separation is important, since it is expected that the two fragments have a different volatility with the inorganic fraction to be linked with NH_4_NO_3_ volatilization indoors [26]. The organic-bonded NO_3_ fraction is given by:

(1) $${\mathrm{pOrgNO}}_{3\;\mathrm{frac}}=\frac{\left(1+R_{{\mathrm{OrgNO}}_{3}}\right)\times \left(R_{\mathrm{measured}}-R_{\mathrm{calib}}\right)}{\left(1+R_{\mathrm{measured}}\right)\times \left(R_{{\mathrm{OrgNO}}_{3}}-R_{\mathrm{calib}}\right)}$$

and the organic-bonded NO_3_ mass concentration by:

(2) $${\mathrm{orgNO}}_{3}={\mathrm{pOrgNO}}_{3\;\mathrm{frac}}\times [{\mathrm{NO}}_{3}]$$

where R_measured_ is the ratio between the signals NO_2_^+^ to NO^+^ (or *m*/*z*46 and *m*/*z*30, respectively) and R_calib_ is the ratio observed during the calibration with ammonium nitrate (here 0.7). The value of R_orgNO3_ was set to be 0.1 according to other studies reporting measurements of organonitrates [45,46]. Moreover, this method is reliable for values of pOrgNO_3 frac_ > 0.15 and orgNO_3_ > 0.1 μg m^−3^ [46]; thus, these thresholds were used to filter out the data (valid data: 77% indoors and 88% outdoors). Subsequently, the inorganic-bonded NO_3_ (inorgNO_3_) can be derived by subtracting orgNO_3_ from the total NO_3_ concentration. 

Analysis of the PTRMS data was performed using PTRMS Viewer (v3.2.7). The first step consisted in recalibrating the mass scale of each acquired spectrum using the large reference peaks at *m*/*z*21.022, 37.0275, 203.943 and 330.848 provided by H_3_O^+^, H_3_O^+^(H_2_O) and the di-iodobenzene internal standard continuously injected in the PTRMS reactor through the built-in Permeation device for Mass Scale Calibration (PerMaSCal) feature. The second step required to generate a peak table containing all the peaks exhibiting an ambient signal statistically larger than the associated background signal. A total of 116 single peaks have been selected over the mass range 21–510 Th. The last step consisted in the deconvolution of all selected peaks using a multi-peak fitting tool. An additional step of filtering the extracted masses was necessary to remove ions generated in the ion source based on their mass, their behavior, and their temporal variability. The final dataset contains 91 ions attributed to ambient traces gases.

### 2.4. Indoor/Outdoor Apportionment Methodology

Each compound can be categorized as originating from indoor, outdoor, or both. In addition, when the outdoor environment is the main source, the indoor environment may either not significantly affect the amount of pollutant transmitted indoor or may act as a sink (through slow chemical processes or adsorption on surfaces). 

For the analysis described below, measurements of PM_1_ and trace gases were averaged over four hours to reduce the impact of the buffering time, which is defined here as the time needed for the indoor environment to respond to an abrupt change in concentration outdoors as previously discussed for the building used in this study [39].

In order to assess the contribution of indoor processes to the observed pollutant concentrations, we assumed perfect instantaneous mixing during air exchange and the invariability of air exchange, penetration and particle deposition throughout the 12-day monitoring period. Under these assumptions, the steady state indoor concentration of a pollutant is given by Equation (3):

(3) $$C_{\mathrm{IN}}=\frac{{\alpha \mathrm{PC}}_{\mathrm{OUT}}}{\alpha +K}+\frac{(\sum^{\;}S)/V}{\alpha +K}$$

where V is the volume of the room (m^3^); C_IN_ and C_OUT_, the indoor and outdoor mass concentrations of a pollutant, respectively; α, the air exchange rate (hr^−1^); P, the penetration factor (dimensionless); K (hr^−1^), either the deposition rate for particles or the first-order loss rate for the reaction of VOCs with gaseous oxidants (OH, O_3_, NO_3_); and ΣS an indoor source/sink term (mass unit per hour) accounting for adsorption/desorption processes leading to either net emission or sink, secondary formation in the gaseous phase (chemical transformations) and any other potential sources or sinks. For instance, if the analysis was focusing on gaseous formaldehyde, the source/sink term ΣS would account for direct emissions from building materials, the release from household chemicals and gas-phase formation from the oxidation of other VOCs.

The first term on the right-hand side of Equation (3) represents the contribution of species with an outdoor origin (Outdoor Contribution: OC), which results from outdoor pollutants partially penetrating indoors through the ventilation system or air leakage through cracks and openings, while the second term on the right-hand side represents the contribution of species with an indoor origin (Indoor Contribution: IC). The indoor concentration of a pollutant can therefore be expressed as follows:

(4) $$C_{\mathrm{IN}}=\mathrm{OC}+\mathrm{IC}\;\mathrm{with}\;\mathrm{OC}={\;F}_{\mathrm{INF}}\times C_{\mathrm{OUT}}$$

The term F_INF_ ($F_{\mathrm{INF}}=\frac{\mathrm{aP}}{a+K}$) is the dimensionless infiltration factor, which represents the penetration factor for outdoor trace gases when chemical reaction rates are considered negligible compared to air exchange, and the fraction of particles that penetrates and remains suspended for outdoor particles. In the following, IC and OC are referred to as indoor and outdoor contributions, respectively.

For this study, infiltration factors were derived for all VOCs as the slope of a linear regression of measured indoor (C_IN_) vs. outdoor (C_OUT_) concentrations, similar to an approach already applied for PM_2.5_ [47]. However, the amplitude of ΣS in Equation (3) can slowly change over time due to changes in environmental conditions, such as temperature, relative humidity, solar irradiation, etc. This change can lead to some scatter in the data when *C*_IN_ is plotted versus C_OUT_, and as a consequence to a bias in the retrieval of F_INF_. In order to minimize this issue, scatter plots were color-coded by time to check whether a time-dependence was observed, and if so, the dataset was binned in time segments (two or more groups of points) where observations indicate that ΣS was not significantly changing. Individual scatter plots related to each group of points showed similar slopes and only the intercepts, which represent average indoor contributions, were different. In this case, a weighted-average value of F_INF_ was derived from the weighted slopes:

(5) $$F_{\mathrm{INF}}=\frac{\sum^{\;}b_{i}N_{i}}{\sum^{\;}N_{i}}$$

where b_i_ are the slopes observed for different groups of points and N_i_ the number of data points.

As shown in Equation (4), the product between F_INF_ and the outdoor concentration of the targeted pollutant provides the contribution of the outdoor environment (OC) to the observed indoor concentration. The contribution of the indoor environment (IC) is given by the difference between C_IN_ and OC. Note that IC and OC are time-dependent and derived with a time resolution of 4 h (averaging time of the indoor/outdoor dataset as mentioned above). 

This methodology is based on the assumption that the indoor reactivity of the targeted pollutants does not significantly impact their ambient concentration. Thus, the slope of the regression line between indoor and outdoor concentrations for a specific trace gas only depends on its penetration factor. However, the reaction rate of some reactive VOCs with gaseous oxidants (OH, O_3_, NO_3_) can compete with air exchange, leading to a breakdown of the assumption (Table S2 in SI). For instance, assuming OH and NO_3_ indoor mixing ratios of 20 ppq and 1 ppt as observed during some studies [48,49,50,51] and a maximum O_3_ mixing ratio of 20 ppb observed in this study, species such as isoprene and monoterpenes are found to break the assumption. This limitation also applies to ozone as discussed in the result section below. Other species including hydrocarbons such as aromatic and OVOCs, such as alcohols, aldehydes, ketones and carboxylic acids, are found to be less reactive, allowing the use of the methodology described above.

Finally, global 4-h time-resolved emission rates were calculated for each VOC considering their indoor contribution:

(6) ER=α×IC

where α is the air exchange rate (hr^−1^) and IC the time resolved indoor contribution (μg m^−3^).

It is interesting to note that the use of the indoor contribution (IC) instead of (C_IN_-C_OUT_) for the calculation of emission rates as performed in other studies [52,53] is more accurate since the potential loss of VOCs in the ventilation system is then taken into account.

## 3. Results

This section first gives an overview of the indoor and outdoor composition and application of the described methodology to calculate the time-resolved IC, OC and ER for the different compounds (VOCs, inorganic gases, PM_1_ species).

### 3.1. Indoor and Outdoor Composition

**Meteorological data**—During the campaign, the temperature ranged between −0.6 and 8.4 °C outdoors and from 8.3 to 14.1 °C indoors (no heating inside the facility), leading to a positive difference of 2.3–11.1 °C, while the relative humidity varied from 68–96% and 43–76% outdoors and indoors, respectively. 

**Gas phase compounds**—Average concentrations for inorganic gases reached 9.3 ± 5.5 ppb and 13.1 ± 6.9 ppb for O_3_; and 11.7 ± 6.9 ppb and 11.2 ± 7.9 ppb for NO_2_ for indoors and outdoors, respectively. Gas phase measurements also included 91 quantified ions (Table S3 in SI reports the exact protonated masses detected by PTRMS and examples of VOCs monitored at these *m*/*z*) both indoors and outdoors, with the exception of 3 ions observed indoors that were below detection limit outdoors. These ions include 20 hydrocarbons and potential fragments of protonated alcohols (C_x_H_y_), and 60 OVOCs which were further separated into three categories given their oxidation state (C_x_H_y_O—*n* = 27, C_x_H_y_O_2_—*n* = 24 and C_x_H_y_O_z≥3_—*n* = 9). From this dataset, 81 molecular formulas were successfully attributed to the detected ions from their exact masses, but 11 masses above 133 Th were not identified due to a lack of mass resolution and are referred to as “Others” hereafter.

**Figure 2:**
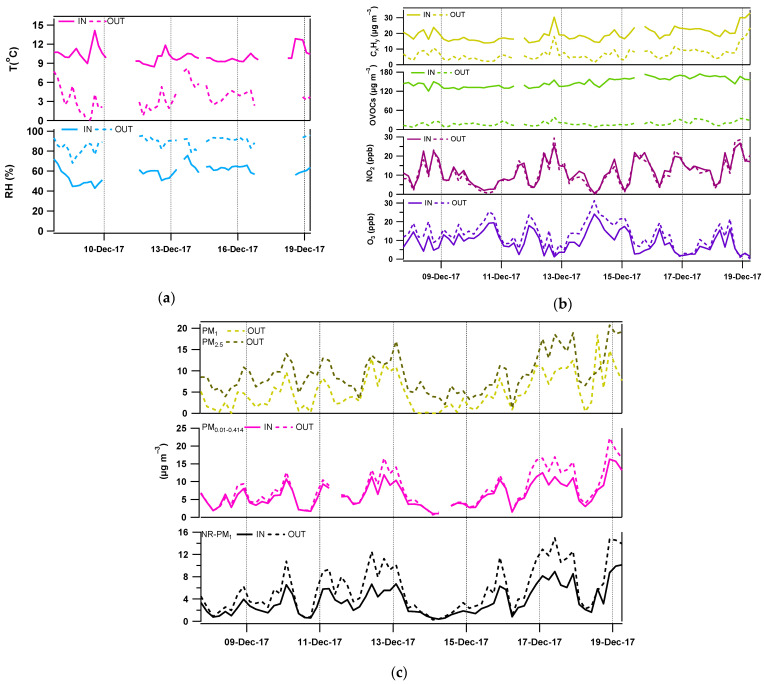
Timeseries of (**a**) meteorological parameters, (**b**) gas phase compounds, and (**c**, from top to bottom) particle mass concentrations monitored by TEOM-FDMS, SMPS and AMS indoors (line) and outdoors (dashed line).

Timeseries of meteorological parameters as well as C_x_H_y_ and OVOCs compounds are shown in Figure 2 (panels a and b, respectively).

**Figure 3:**
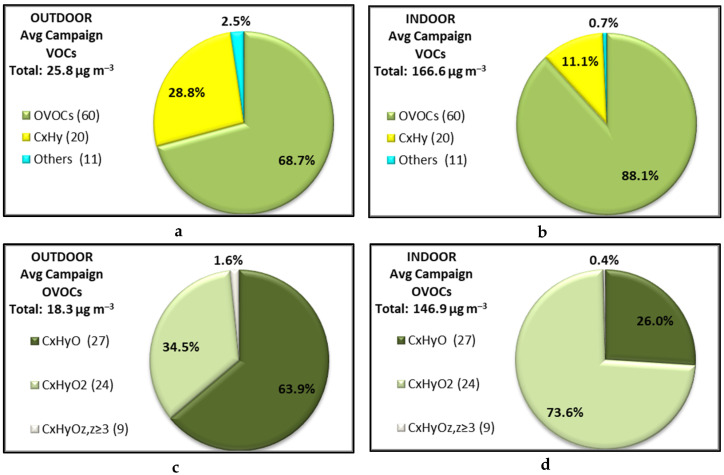

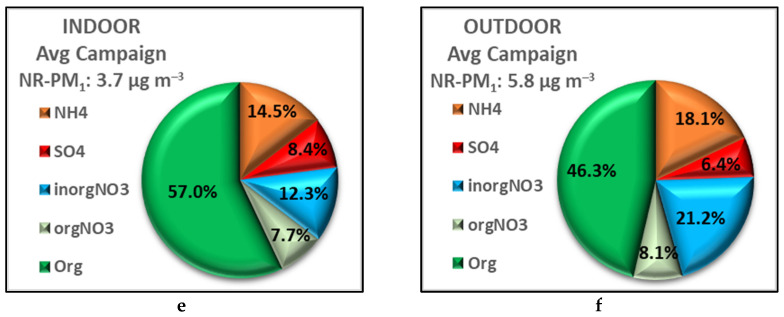
(**a**,**b**) Contributions of OVOCs (green), hydrocarbons and potential fragments of alcohols (C_x_H_y_, yellow) and others (light blue) to the observed pool of VOCs (**a**) outdoors and (**b**) indoors; (**c**,**d**) contributions of C_x_H_y_O (dark green), C_x_H_y_O_2_ (light green) and C_x_H_y_O_z≥3_ (white) compounds to the total pool of observed OVOCs (**c**) outdoors and (**d**) indoors. Values in parenthesis indicate the number of measured species. (**e**,**f**) Contribution of inorganic (NH_4_: orange, inorgNO_3_: light blue, orgNO_3_: light green, SO_4_: red) and organic (green) fractions to the total mass of NR-PM_1_ particles (**e**) indoors and (**f**) outdoors.

On average, OVOCs contributed 88% to the total observed VOC concentration indoors, while only 69% outdoors. Conversely, hydrocarbons contributed 11% indoors compared to 29% outdoors (Figure 3a,b). The contribution of unidentified VOCs (categorized as Others) was lower than 3% for both indoors and outdoors. The total mass of VOCs increased from 26 to 167 μg m^−3^ when ambient air was transferred indoors, a change which is mainly due to an increase of OVOCs by a factor of 8 and an increase of C_x_H_y_ by a factor of 3. It should be noted that this comparison likely underestimates the contribution of hydrocarbons to the total pool of VOCs since the PTRMS technique is blind to alkanes, which have been observed at significant concentrations indoors [54,55]. More details for each species are given in Table S4. 

Previous studies have also reported high concentrations of VOCs in various indoor environments (school classrooms, offices, houses/apartments) [28,32,52,56,57,58,59,60,61,62,63,64,65,66,67,68,69]. Interestingly, the concentrations observed in unoccupied buildings are elevated, often reaching similar or even higher levels than under occupied conditions. The wood structure of the facility does not lead to a significant increase of isoprene or monoterpenes compared to other buildings. 

Figure 3 also reports the contribution of OVOCs outdoors (panel c) and indoors (panel d) grouped by category (C_x_H_y_O, C_x_H_y_O_2_, C_x_H_y_O_z≥3_). It is evident that while the most abundant compounds outdoors are those with one atom of oxygen, the most abundant compounds indoors contain two oxygen atoms, with the C_x_H_y_O_2_ contribution to the total OVOCs more than doubling indoors. In the latter category, acetic (C_2_H_4_O_2_.H^+^) and formic (CH_2_O_2_.H^+^) acids contribute 44% and 13%, respectively, to the total concentration of VOCs, while their respective contributions outdoors are 13% and 4%. These findings imply that there is a strong source of carboxylic acids indoors, possibly being emitted by building materials and/or produced by chemical transformations, accounting for most of the OVOC mass, in agreement with previous studies [56,70,71].

**Particulate Matter**—Concentrations of particles in the size range 10.2–414 nm (from SMPS measurements) ranged from 0.98–16.3 µg m^−3^ and 0.70–22.2 µg m^−3^ indoors and outdoors, respectively, showing a similar temporal variability in both environments, though in lower concentrations indoors by approximately 13%. Moreover, the NR-PM_1_ indoor concentrations are usually lower than outdoor concentrations, showing mean Indoor-to-Outdoor ratios (I/O) below unity, in agreement with previous studies [27,72]. More specifically, Figure 3 (panels e and f) depicts the contribution of inorganic (NH_4_: orange, inorgNO_3_: light blue, SO_4_: red), organic (green) and orgNO_3_ (light green) fractions to the total mass. Total concentration of NR-PM_1_ has decreased from 5.8 μg m^−3^ outdoors to 3.7 μg m^−3^ indoors (Figure 3e,f). The organic and sulfate contributions increased by approximately 10% and 2% from outdoors to indoors, while nitrate (mainly the inorganic bonded nitrate, inorgNO_3_) and ammonium contributions decreased by 9% and 4%, respectively. Finally, particles measured by the optical counters (OPC, 0.3 to 10.0 μm) followed a similar behavior showing lower concentrations indoors compared to outdoors (I/O ratio 0.7 for the smaller fraction, down to 0.14 for larger particles). Descriptive statistics for each particle species are presented in Table S5.

Previous studies conducted in a classroom, a guest and residential house and a multi-use building [26,27,31,72] reported concentrations outdoors in the range of 0.86–4.0 μg m^−3^, 0.44–3.2 μg m^−3^, 1.35–7.2 μg m^−3^, and 3.24–8.2 μg m^−3^ for SO_4_, NH_4_, NO_3_ and Org, respectively, while indoors the respective concentrations were 0.28–2.0 μg m^−3^, 0.05–0.9 μg m^− 3^, 0.04–1.4 μg m^−3^ and 1.14–4.4 μg m^−3^, being similar to that observed in our study apart from SO_4_ and Org outdoors which are slightly lower.

### 3.2. Contribution of the Indoor Environment to Observed Gaseous Pollutant Concentrations

The Indoor-to-Outdoor (I/O) ratio is only a primary indicator for the existence (or not) of indoor sources. In order to decouple the contributions of indoor and outdoor sources to observed indoor pollutant concentrations, the methodology described in Section 2.4 was applied using Equations (3) and (4). 

**Figure 4:**
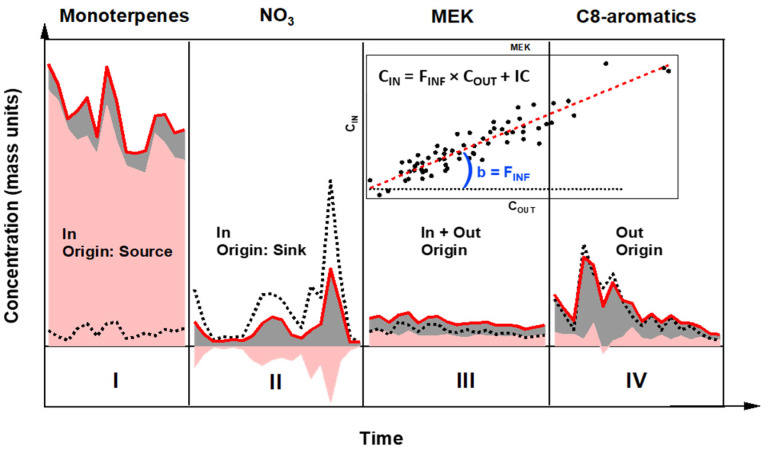
Schematic of possible indoor (IC; shaded pink area) and outdoor (OC; shaded grey area) contributions for four cases, calculated from indoor (red solid line) and outdoor (black dotted line) mass concentrations. Gas phase: panels I, III, IV. Particle phase: panel II. Inset plot: example of scatter plot for the retrieval of the infiltration factor (species: MEK).

The results of this study varied depending on the targeted pollutant. A compound exhibiting a large IC value (Figure 4, panel I) is categorized as a compound of indoor origin, while one originating from both indoors and outdoors will exhibit IC and OC values of the same magnitude (panel III). When the outdoor environment is the main source (panels II and IV), the indoor environment might act as a sink due to chemical reactivity or adsorption on surfaces for gases (IC < 0; panel II) or might not significantly impact the pollutant levels (indoor and outdoor concentrations exhibiting close covariation; panel IV).

**Figure 5:**
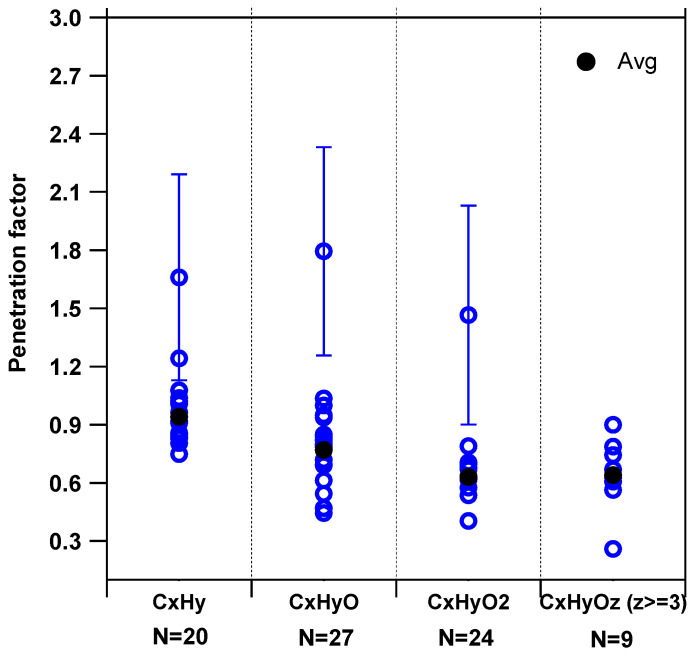
Penetration factors derived for different categories of VOCs. Average values are shown in black. Error bars represent 3σ. N is the number of species reported for each category.

**VOCs**—Figure 5 displays penetration factors for all monitored ions (blue), derived using the methodology described in Section 2.4 as a function of the four categories of VOCs defined above (C_x_H_y_, C_x_H_y_O, C_x_H_y_O_2_ and C_x_H_y_O_z≥3_). For this analysis, only scatter plots of C_IN_ vs. C_OUT_ exhibiting statistically significant slopes, i.e., a 3σ relative standard deviation (RSD) lower than 50%, were accepted. As can be observed from this figure, the penetration efficiency decreases from approximately 0.9 (C_x_H_y_) to 0.7 (C_x_H_y_O_3_) as the number of oxygens in the molecule increases. This observation is likely due to a decrease in VOC volatility when the number of oxygen atoms increases, which translates into a higher “stickiness” on filters located in the ventilation system, especially for OVOCs in contact with humid surfaces since most of them are water soluble [73]. While three compounds or group of isomers (C_10_H_16_, C_9_H_14_O, C_7_H_14_O_2_) seem to exhibit a penetration factor higher than unity, the lower bounds (considering an uncertainty at 3σ) are close to unity. As mentioned above, the use of a C_IN_-C_OUT_ scatter plot may not reliably provide the penetration factor if the compound of interest exhibits a significant reactivity with ambient oxidants (competition with air exchange). It is interesting to note that C_10_H_16_ compounds are likely monoterpenes, for which the assumption of low reactivity indoors breaks down (Section 2.4). In addition, oxidation products from monoterpenes are expected to be observed as C_9_H_14_O compounds (limonaketone, nopinone). The larger-than-expected penetration factors observed for C_9_H_14_O and C_7_H_14_O_2_ compounds are therefore likely due to an impact of chemical processes on the slopes of the C_IN_-C_OUT_ scatter plots.

**Figure 6:**
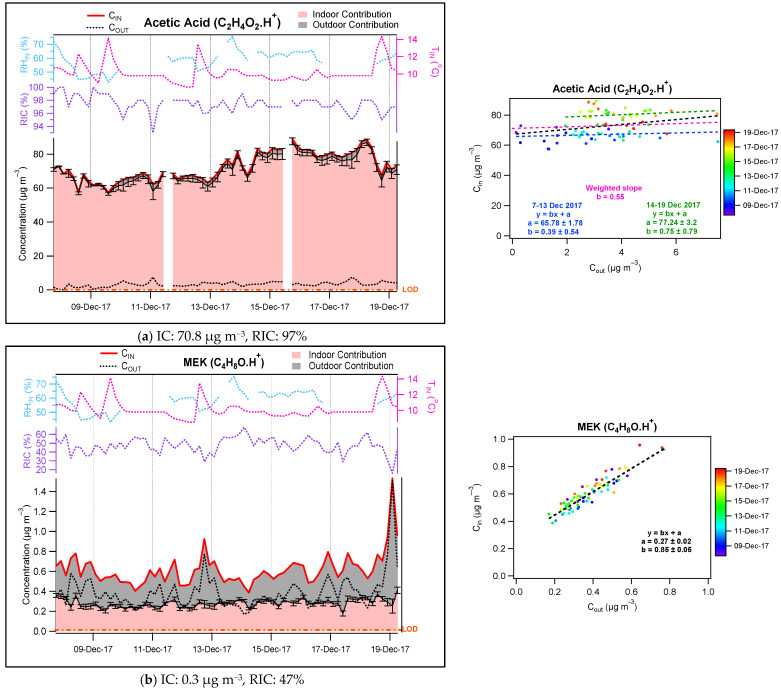

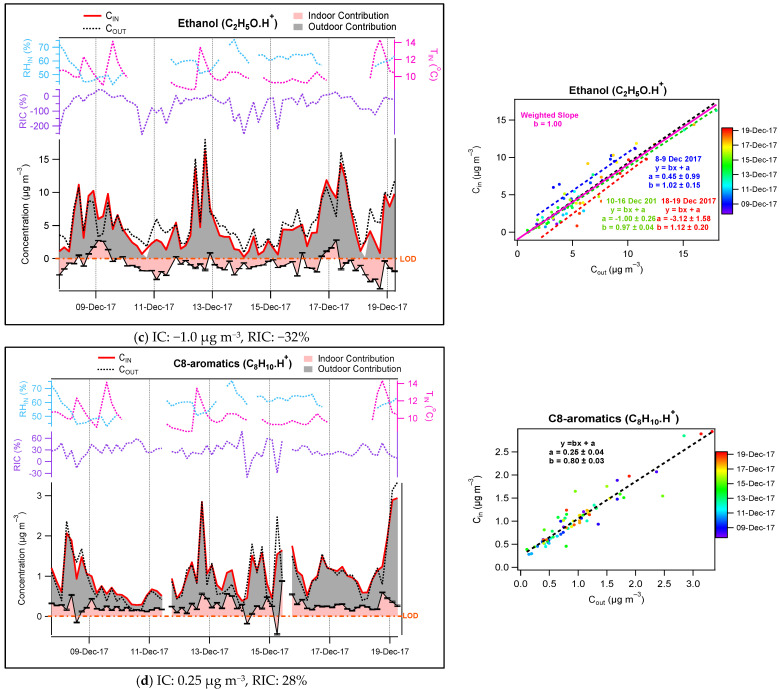
(**a**–**d**) Examples of IC-OC contributions (left) and scatter plots of indoor-outdoor concentrations (right). Error bars represent 1σ.

Example of scatter plots between measured indoor and outdoor concentrations, color-coded according to the date of the measurements, are shown in Figure 6 for selected species exhibiting different origins and behaviors (a: main indoor origin, b: both indoor and outdoor origins, c: main outdoor origin and indoor environment acting as a sink, d: main outdoor origin). For instance, the scatter plot for MEK (detected as C_4_H_8_O.H^+^ in PTRMS) shown in panel b leads to a slope of 0.85 ± 0.05 (1σ), which represents the penetration factor for this compound, and a y-intercept of 0.27 ± 0.02 (1σ) µg m^−3^, which provides an average value of its IC over the whole campaign. As shown on panel a, a precise penetration factor cannot be derived for compounds exhibiting a large y-intercept such as acetic acid (C_2_H_4_O_2_.H^+^) since they show poor correlation with outdoor concentration and their main source is the indoor environment. 

Figure 6 also shows concentrations measured indoors (red line) and outdoors (black dashed line), together with the indoor (pink fill) and outdoor (grey fill) contributions for the selected compounds. In addition, the relative indoor contribution (RIC = IC/C_IN_, purple dashed line), relative humidity (RH, blue dashed line) and temperature (T, pink dashed line) measured indoors are also plotted, together with the PTRMS detection limit (LOD, orange dashed line). As mentioned above, four main categories of species exhibiting different origins and behaviors can be discussed separately: 

(i) A compound characterized by a much higher indoor than outdoor contribution (typically an average RIC over 70%) is categorized as a compound of indoor origin (Figure 6a). Acetic acid (C_2_H_4_O_2_.H^+^) is one such compounds exhibiting this behavior with an average indoor contribution of 70.8 μg m^−3^ (RIC of 97%).

(ii) A compound is categorized as originating from both indoors and outdoors (Figure 6b) when the average RIC is between 30% and 70%. C_4_H_8_O.H^+^ (tentatively identified as methylethylketone) exhibits an average RIC of 47% (IC = 0.3 µg m^−3^), indicating a similar impact of both environments on indoor concentrations.

(iii, iv) Compounds originating mainly from outdoors (average IC below 30%; Figure 6c,d). For these species, indoor concentrations can be highly variable since it mainly depends on the OC and as a consequence on fast varying outdoor sources. This case can be further divided into two categories depending whether the indoor environment acts as a sink (IC < 0) or does not significantly impact the concentration of the infiltrated compound (IC ≈ 0). 

(iii) For instance, the IC for ethanol (C_2_H_5_O.H^+^; Figure 6c) is negative most of the time, with approximately 32% of this compound lost, either through chemical processes (reaction with oxidants) or adsorption on surfaces. Since ethanol reacts slowly with oxidants but is known to stick easily on surfaces, adsorption processes are likely the cause for its indoor sink. Since the adsorption rate depends on the indoor VOC concentration, this process will likely lead to an underestimation of the penetration factor that is derived from the C_IN_-C_OUT_ scatter plot, which in turn may lead to an underestimation of the sink effect (overestimation of IC).

(iv) The example of C8-aromatics (C_8_H_10_.H^+^; Figure 6d) is also interesting because the time series of indoor and outdoor concentrations are very close and covary together over time, suggesting that these compounds are only of outdoor origin. However, the same figure also shows that the indoor environment contributes to approximately 30% of the indoor concentration. These contrasting observations are likely due to a compensation between a penetration factor lower than unity and the presence of small indoor emissions. 

**Figure 7:**
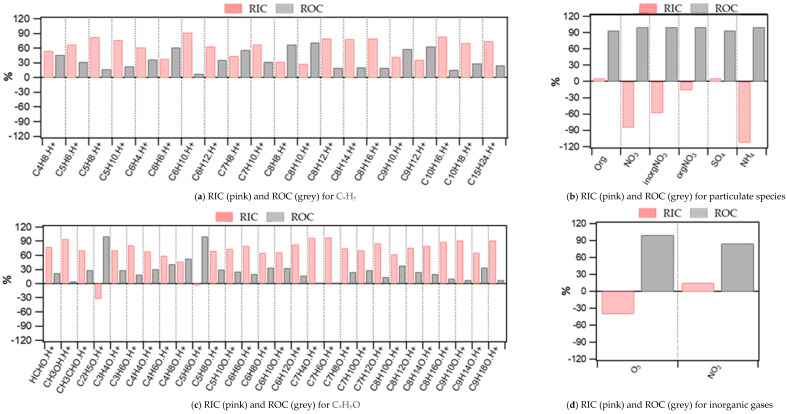

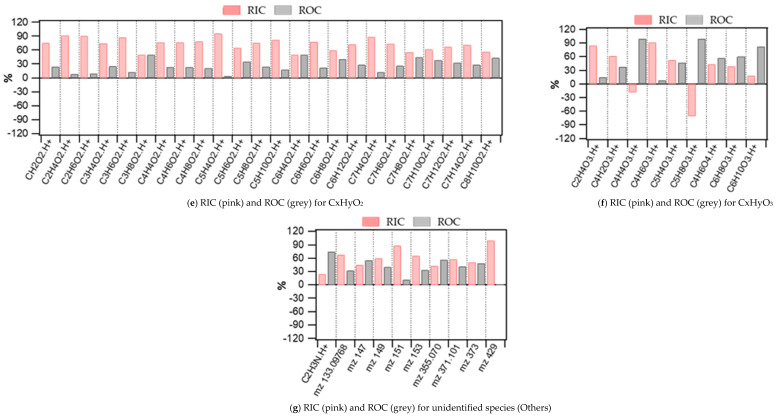
(**a**–**g**) Average relative indoor and outdoor contributions (RIC and ROC, respectively) for all compounds during the intensive campaign.

The average relative indoor (RIC, in pink) and outdoor (ROC, in grey) contributions for all compounds measured during the intensive campaign are displayed in Figure 7. They are also reported in Table S6, together with the slopes of C_IN_-C_OUT_ scatter plots and absolute indoor contributions (IC).

**Figure 8:**
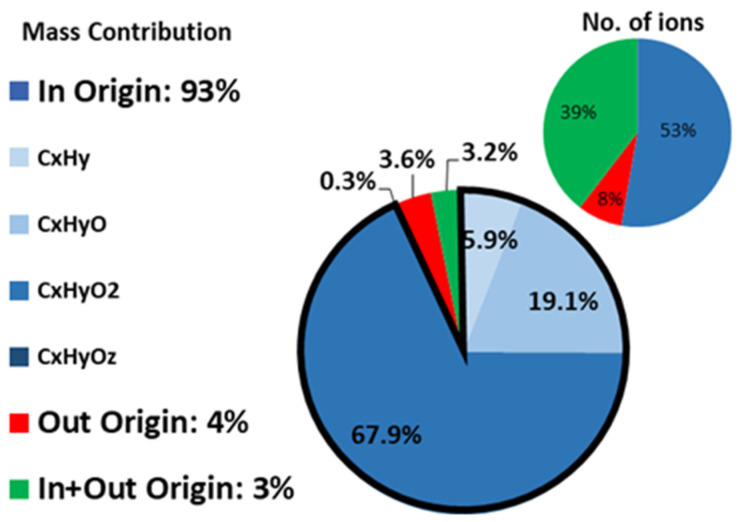
Origin of VOCs (grouped by category) observed indoors in terms of number of ions (small pie) and of mass concentration (large pie).

Considering all the ions detected by PTRMS, the majority of them (53%) originates from indoors (RIC > 70%), corresponding to 93% of the total indoor mass of measured VOCs. The other half of the compounds is divided between compounds which had the outdoor air as a main source (8% of the compounds with RIC < 30%), holding 4% of the total indoor mass, and compounds that had both outdoor and indoor contributions (39% of the compounds, 30% < RIC < 70%), holding 3% of the total indoor mass (Figure 8). As shown in Figure 8, a large mass contribution (68%) for species of indoor origin comes from compounds containing two atoms of oxygen, which likely include a large number of carboxylic acids. Indeed, formic acid (CH_2_OH.H^+^) and acetic acid (C_2_H_4_O_2_.H^+^) contribute 20% and 68%, respectively, to the total indoor concentration of this family. High concentrations of carboxylic acids have also been previously reported by Liu et al. [56] in a university classroom, indicating that formic acid is emitted by wood-based products or latex paint, which are two materials that were present in our building. In addition, large concentrations of OVOCs observed in our study (methanol) have also been observed in other types of buildings and are actually in the lower range of reported values [28,30]. These results tend to indicate that the building used in our study has a chemical signature similar to that of other buildings previously used to investigate indoor air quality.

These results show that the indoor environment acts mainly as a source of VOCs by emitting these compounds continuously, even in the absence of occupants and furnishing. Average emission rates (ER; Equation (6)) were found to be 0.20, 0.19, 0.14, 0.03 and 0.04 μg m^−3^ h^−1^ for C_x_H_y_, C_x_H_y_O, C_x_H_y_O_2_, C_x_H_y_O_z≥3_ and unidentified species, respectively. A few species have been observed to exhibit high emission rates amongst the abovementioned VOC categories. For instance, methanol (CH_3_OH.H^+^) has also been reported in previous studies and attributed to emissions from wood decomposition [74]. Formic (CH_2_OH.H^+^) and acetic acids (C_2_H_4_O_2_.H^+^) are emitted as well by wood-based products and wood decomposition or latex paint [56,74]. C_5_H_4_O_2_.H^+^ is tentatively identified as furfural, which has been reported in previous studies as emitted by wood products too [56,75]. Overall emission rates observed in this study are inside the observed range of previous studies [33,76].

**Figure 9:**
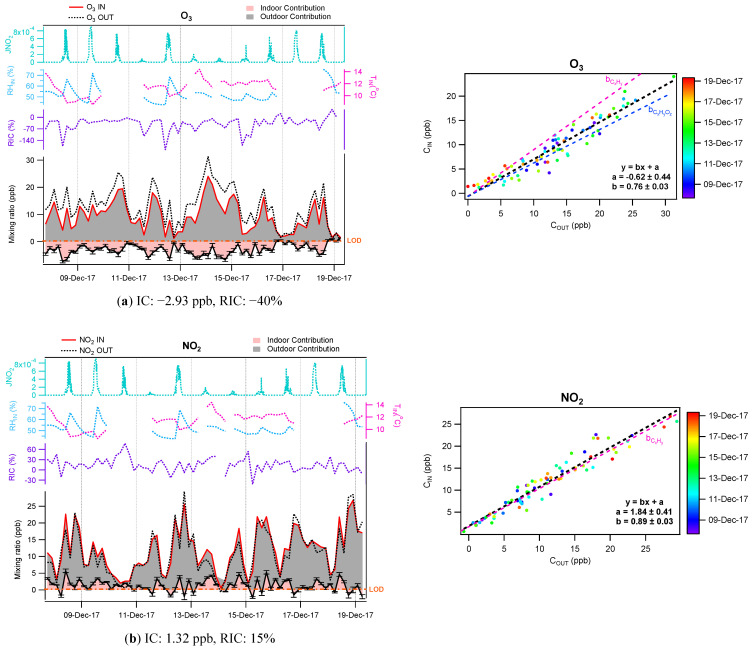
IC-OC contributions (**left**) and scatter plots of indoor-outdoor concentrations (**right**) for (**a**) O_3_ and (**b**) NO_2_. Error bars represent 1σ.

**Inorganic gases**—Indoor and outdoor contributions were also inferred for both O_3_ and NO_2_. The scatter plots between indoor and outdoor concentrations shown in Figure 9 show a small dispersion around the regression line, indicating some variability in the strength of their indoor sources and sinks, however not significantly larger than for VOCs reported in Figure 6. The penetration factors of approximately 0.9 and 0.7 observed for C_x_H_y_ and C_x_H_y_O_z_ species are also reported on these scatter plots for comparison. It can be seen that the slope observed for NO_2_ is very close to the penetration factor characteristic of C_x_H_y_ species, which seems to indicate that the slope is not affected by significant indoor chemistry, while for O_3_, the slope of the scatter plot is closer to that observed for C_x_H_y_O_z_ species. The lower slope for O_3_ could be either due to fast indoor reactions involving ozone or a loss of ozone into the ventilation system. In order to perform the IC-OC analysis for O_3_, the penetration factor observed for C_x_H_y_ species was used in the calculations assuming that O_3_ is lost through indoor reactivity. This penetration factor has to be seen as an upper limit due to possible losses of O_3_ in the ventilation unit, which in turn will lead to a lower limit (respectively, an upper limit) of IC if its sign is positive (source) (respectively, negative (sink)). 

As can be seen in Figure 9, the indoor environment acts mainly as a sink for O_3_ and as a small source for NO_2_, with RIC of −40% and +15%, respectively. Interestingly, both O_3_ and NO_2_ are known to be linked together through the O_3_-NO_x_ photostationary state (PSS) in the atmosphere (Reactions 7–9) [77].

O_3_ + NO → NO_2_ + O_2_ (7)

NO_2_ + hν → NO + O(^3^P) (8)

O(^3^P) + O_2_ + M → O_3_ + M (9)

During daytime, this PSS is disturbed when ambient air is transferred indoors since the photolysis frequency of NO_2_ is larger outdoors than indoors. When the air enters the room, the photolysis rate of NO_2_ (Reaction 8) decreases and, as a result, the PSS switches towards the formation of NO_2_. This effect will appear indoors as a source of NO_2_ and a sink of O_3_. According to reactions 7–9, the conversion between O_3_ and NO_2_ should be 1:1. However, the IC-OC analysis revealed a larger decrease of ozone compared to the increase of NO_2_ indicating that a significant amount of O_3_ is lost indoors, likely through its reaction with unsaturated species in the gas-phase and heterogeneous reactions on surfaces.

### 3.3. Contribution of the Indoor Environment to the Observed Concentrations of Submicron Particulate Species 

The contribution of the indoor environment to PM species concentrations may arise from indoor emissions but also physicochemical processes, such as volatilization, condensation and chemical transformations, which can impact the slope of the C_IN_-C_OUT_ scatter plot. The slope of the scatter plot provides in the case of particles the infiltration factor, which is different from the penetration factor since deposition can be significant. Unlike trace gases, the RIC in the case of particles was normalized to OC to avoid large uncertainties related to low concentrations that are sometimes observed indoors.

**Figure 10:**
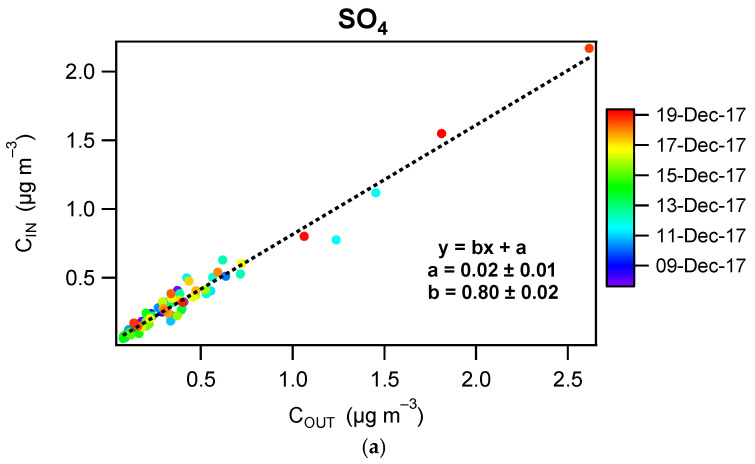

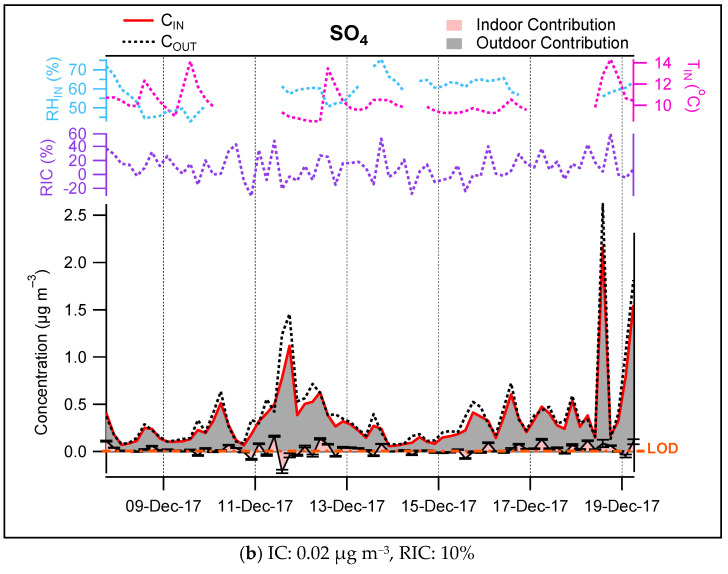
(**a**) Scatter plot of indoor-outdoor concentrations color-coded by date for NR-PM_1_ SO_4_, (**b**) IC-OC contribution. Error bars represent 1σ.

**SO_4_**—For sulfate, a low level of dispersion around the regression line and an intercept close to zero is observed on the scatter plot shown in Figure 10 (panel a). The penetration factor derived for SO_4_ from the scatter plot is 0.80, which is consistent with the penetration factor of 0.75 measured for PM_1_ particles (Figure S2 in SI). This agreement is expected considering the absence of both (i) known sources of SO_4_ indoors [27] and (ii) physicochemical processes that would significantly affect its concentration. As a consequence, the indoor contribution of sulfate was found to be close to zero (IC = 0.02 μg m^−3^, Figure 10b). Since the behavior of SO_4_ only reflects the impact of mechanical losses, the infiltration factor determined for this species was used below to infer indoor contributions for other components of NR-PM_1_ (NO_3_, NH_4_, Org) and to assess whether additional mass losses due to volatilization and other physicochemical transformations are occurring for these species (Figure S5 in SI). 

**Figure 11:**
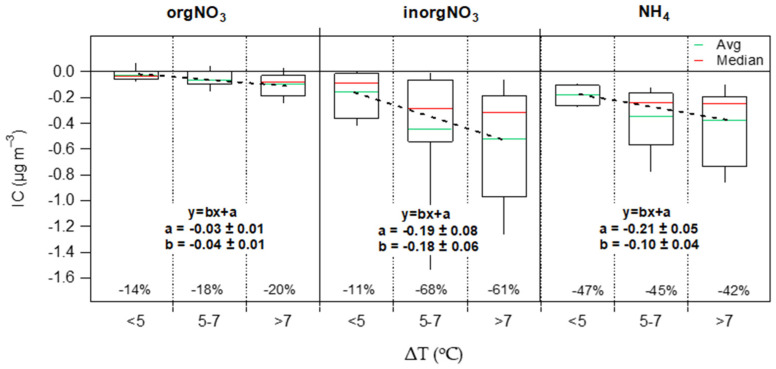
Indoor contribution of orgNO_3_, inorgNO_3_ and NH_4_ as a function of indoor-outdoor temperature differences. The boxes and whiskers (**bottom** to **top**) represent the 10th and 25th percentiles, median (red line), and the 75th and 90th percentiles. Green lines represent average IC. Percentages correspond to the relative indoor contribution (RIC = IC/OC) for each bin.

**NH_4_ and NO_3_**—The RIC of nitrate (orgNO_3_ and inorgNO_3_) and ammonium were found to be −19%, −57% and −47%, respectively (Figure S6 in SI), clearly showing that the indoor environment acts as a sink for both NH_4_ and NO_3_. This behavior is likely due to the volatilization of NH_4_NO_3_ when ambient air enters into a warmer environment. As shown in Figure 11, the average RIC of NH_4_, inorgNO_3_ and orgNO_3_ decreased by 6%, 50% and 5%, respectively, as the difference in temperature between indoors and outdoors (ΔT) increased from at least 2 °C (<5 °C to >7 °C). These results indicate a higher volatilization rate for inorgNO_3_ compared to orgNO_3_ since the latter is less volatile than the inorganic fraction. This result, which has also been observed by Avery et al. [26], not only corroborates the IC/OC calculation method, but independently verifies as well the orgNO_3_ estimation from AMS [45].

**Figure 12:**
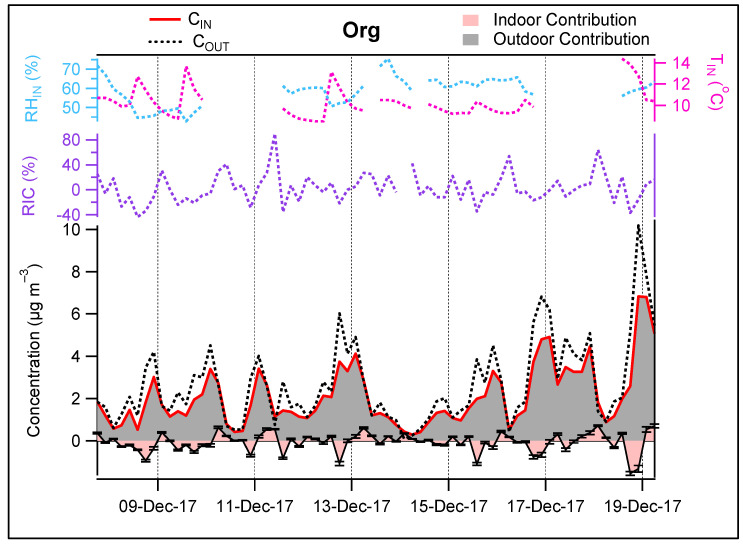
IC-OC contribution of Org. IC: −0.03 μg m^−3^, RIC: 3%. Error bars represent 1σ.

**Organics**—The average RIC for the organic fraction was found to be 3% ranging from −34% to 65% (Figure 12). 

**Figure 13:**
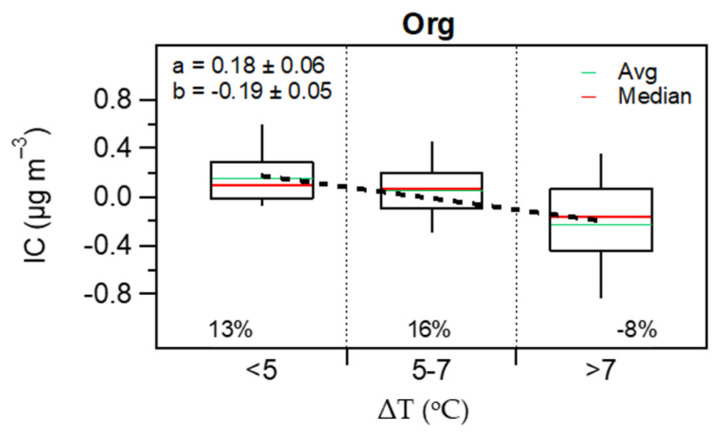
Indoor contributions of Organics as a function of indoor-outdoor temperature differences. The boxes and whiskers (bottom to top) represent the 10th and 25th percentiles, median (red line), and the 75th and 90th percentiles. Green lines represent average IC. Percentages correspond to the relative indoor contribution (RIC = IC/OC) for each bin.

The RIC dependency on the indoor-outdoor temperature gradient showed a decrease from 13% (enrichment in organics) to −8% (depletion) for the total organic fraction when ΔT increases from <5 °C to >7 °C (Figure 13). According to Avery et al. [26], many families of organics and their gas-phase precursors are water soluble; thus, these species will be more likely lost when passing through the ventilation system which contains aqueous films. This is consistent with previous observations from this work for VOCs (Figure 5), where the penetration efficiency of the oxygenated VOCs decreased as the number of oxygens increased in the molecule.

### 3.4. Method Limitations

While the methodology described here performs well for the majority of the targeted pollutants, it is important to note that there are also some important limitations. A crucial step in the proposed methodology is to derive a penetration factor for each species of interest from the slope of a linear regression between its measured indoor and outdoor concentrations. The retrieval of the penetration factor can be biased by physicochemical processes operating indoors. Those include the reactivity with ambient oxidants (OH, O_3_ and NO_3_) for VOCs and physicochemical transformations for particles, if the rate of these processes compares with that of the air exchange. For example, it was found that oxidants such as O_3_, and reactive VOCs, such as isoprene and monoterpenes, cannot be reliably analyzed with the proposed methodology. A similar concern could be expressed for other VOCs exhibiting a significant reactivity with ambient oxidants, which unfortunately could not be investigated in this study since Time-of-Flight PTRMS measurements only provide information on the elemental composition of detected ions and no information on their chemical structure and reactivity. It is therefore recommended to carefully apply this methodology with online measurements from instruments that can provide molecular structure information (e.g., gas chromatography). For particles, this issue can be avoided when the chemical composition is monitored since the infiltration factor observed for non-reactive species such as SO_4_ can be used for the analysis of all particulate species.

Furthermore, although this methodology can highlight trace gases lost indoors (negative IC due to adsorption processes), the strength of the sink will likely be underestimated due to the impact of the loss processes on the retrieval of the penetration factor, as discussed for ethanol.

Finally, this methodology is not considered applicable for compounds exhibiting low concentrations indoors, for instance, OVOCs potentially impacted by their formation from fast homogeneous or heterogeneous chemical reactions, or semi-volatile compounds impacted by a shift in gas/particle partitioning induced by temperature gradients from outdoors/indoors.

## 4. Conclusions

A simple apportionment methodology has been described in detail in this study and was applied to a vacant facility, typical of an energy-efficient building, to assess whether and how the unoccupied indoor environment affects the chemical composition of infiltrated ambient air. Concomitant indoor and outdoor time-resolved measurements of inorganic trace gases, VOCs and NR-PM_1_ particles (including composition, i.e., SO_4_, NH_4_, inorgNO_3_, orgNO_3_ and Org) from online mass spectrometers were used to categorize the indoor/outdoor nature of the targeted pollutants. This methodology provided an apportionment of indoor and outdoor contributions to observe pollutant levels, identify whether the indoor environment acts as a source or a sink, and provide information on physicochemical processes operating indoors.

The results indicate that the indoor environment mainly acts as a source for VOCs, leading to a large enrichment in these species, by approximately a factor of 3 and 17 for OVOCs containing 1 and 2 atoms of oxygen, respectively. An increase in concentration was observed for nine C_x_H_y_, 18 C_x_H_y_O, 15 C_x_H_y_O_2_ and two C_x_H_y_O_z_ species, the indoor environment acting as a sink for only a few compounds, including ethanol and a few other OVOCs (corresponding to C_5_H_6_O.H^+^, C_4_H_4_O_3_.H^+^ and C_5_H_8_O_3_.H^+^ ions in the PTRMS). Unsurprisingly, the indoor environment acts as a sink for O_3_ due to its high reactivity with alkenes present in ambient air or adsorbed on surfaces. In contrast, the NR-PM_1_ concentration is reduced by a factor of approximately 2 on average when ambient air is transferred indoors, with different reductions in SO_4_ (factor of 1.2), NH_4_ (1.9), NO_3_ (2.3) and Org (1.3). This analysis also provided evidence that in addition to direct emissions, the unoccupied indoor environment impacts the chemical composition of infiltrated air through changes in the O_3_-NO_x_ photostationary state and physicochemical processes associated with the volatilization of ammonium nitrate.

## Figures and Tables

**Table 1:** Summary of instrumentation, time resolution and limit of detection (LOD).

Instrument (Manufacturer)	Measured Parameters or Species	Time Resolution	LOD (3σ)		Location
AEROTRAK Handheld Particle Counter 8220 (TSI)	Particle number concentrations (6 size bins: 0.3–5.0 μm)	50 s	n/a		IN, OUT
HR-ToF-AMS (Aerodyne Research)	Non-refractory PM_1_: NO_3_, SO_4_, Cl, NH_4_ and Organics	5 min	Species *	V mode (μg m^−3^)	IN, OUT
			Org	0.64	
			SO_4_	0.05	
			NO_3_	0.28	
			NH_4_	0.37	
			Cl	0.19	
PTR—QiToFMS (Ionicon Analytik)	VOCs	10 s	*m*/*z* 79: <30 ppt *m*/*z* 205: <1 ppt		IN, OUT
CO_2_ analyzer (Horiba)	CO_2_	1 min	0.2 ppb		IN, OUT
NO_2_ CAPS (Aerodyne Research)	NO_2_	10 s	0.1 ppb		IN, OUT
O_3_ analyzer (Environnement SA)	O_3_	10 s	0.4 ppb		IN, OUT
CO_2_, RH, T probes (Testo)	CO_2_, Relative Humidity, Temperature	20 s	n/a		IN
TEOM FDMS (Thermo Fisher Scientific, Waltham, MA, USA)	PM_1_ mass concentrations	1 h	n/a		OUT
SMPS (TSI, model 3788)	Particle number concentrations (10.2–414.2 nm)	4.5 min	n/a		IN, OUT
Spectroradiometer (METCON)	Photolysis rate of NO_2_ (J_NO2_)	1 s	n/a		IN

* LOD has been calculated as the 3σ of the daily 10-min blank measurements.

## Data Availability

The data presented in this study are available on request from the corresponding author.

## Supplementary Materials

The following supporting information can be downloaded at: https://www.mdpi.com/article/10.3390/toxics10040161/s1, Glossary. Figure S1: Schematic of the measurement facility: (a) front side, (b) back side, (c) floor plan of the instrumentation room (left) and the experimental room (right). Figure S2 Average values of penetration factors during the intensive campaign as a function of particle size. Figure S3: Schematic of (a) AMS calibration unit and (b) AMS sampling configuration coupled with the CPC, SMPS and CO_2_ analyzer. Figure S4: Schematic of the PTR-MS configuration coupled with the O_3_ and NO_2_ analyzers. Figure S5: Scatter plots of C_IN_ vs. C_OUT_ for NH_4_, NO_3_, and Org. Figure S6: IC-OC contributions (a) orgNO_3_, (b) inorgNO_3_ and (c) NH_4_. Table S1: Compounds in the gas standard used for the transmission curve determination. Table S2: Calculations of VOC oxidation rates and comparison to the air exchange rate (a = 0.54 h^−1^). Table S3: Exact protonated masses detected by PTRMS and examples of VOCs monitored at these *m*/*z*. Table S4: Descriptive statistics of compounds measured by PTRMS. Table S5: Descriptive statistics of measured particles (mass and number concentration). Table S6: Parameters from C_IN_ vs. C_OUT_ scatter plots, indoor (IC) and outdoor contributions, (OC), and emission rates (ER) for aerosols and trace gases.
