# Supplementary material for: Using Real Time Measurements to Derive the Indoor and Outdoor Contributions of Submicron Particulate Species and Trace Gases
Source: Toxics. 2022 Mar 29;10(4):161. doi: 10.3390/toxics10040161 (PMC9024529; doi:10.3390/toxics10040161)
Supplement: Supplementary file 1 [file toxics-10-00161-s001.zip › toxics-1645809-supplementary.pdf]

toxics

Supplementary Materials: Using Real Time Measurements to Derive the Indoor and Outdoor Contributions of Submicron Particulate Species and Trace Gases

Evdokia Stratigou, Sébastien Dusanter, Joel Brito, Emmanuel Tison and Véronique Riffault

	**GLOSSARY**
VOC	Volatile Organic Compound
PM	Particulate Matter
AMS	Aerosol Mass Spectrometer
CPC	Condensation Particle Counter
CxHy	Hydrocarbon
$C_xH_yO_n$	OVOC with n atoms of oxygen
DMA	Differential Mobility Analyzer
ER	Emission Rate
FDMS	Filter Dynamic Measurement System
I/O	Indoor to Outdoor ratio
IC	Indoor Contribution
IE	Ionization Efficiency
inorgNO_3	Inorganic bonded nitrate
LOD	Limit Of Detection
NR-PM_1	Non Refractory PM_1
OC	Outdoor Contribution
OPC	Optical Particle Counter
orgNO_3	Organic bonded nitrate
OVOC	Oxygenated Volatile Organic Compound
PSS	Photostationary State
PTRMS	Proton Transfer Mass Spectrometer
RIC	Relative Indoor Contribution
RIE	Relative Ionization Efficiency
ROC	Relative Outdoor Contribution
SMPS	Scanning Mobility Particle Sizer
SV	Solenoid Valve
TEOM	Tapered Element Oscillating Microbalance
TOC	Total Organic Carbon
WHO	World Health Organization

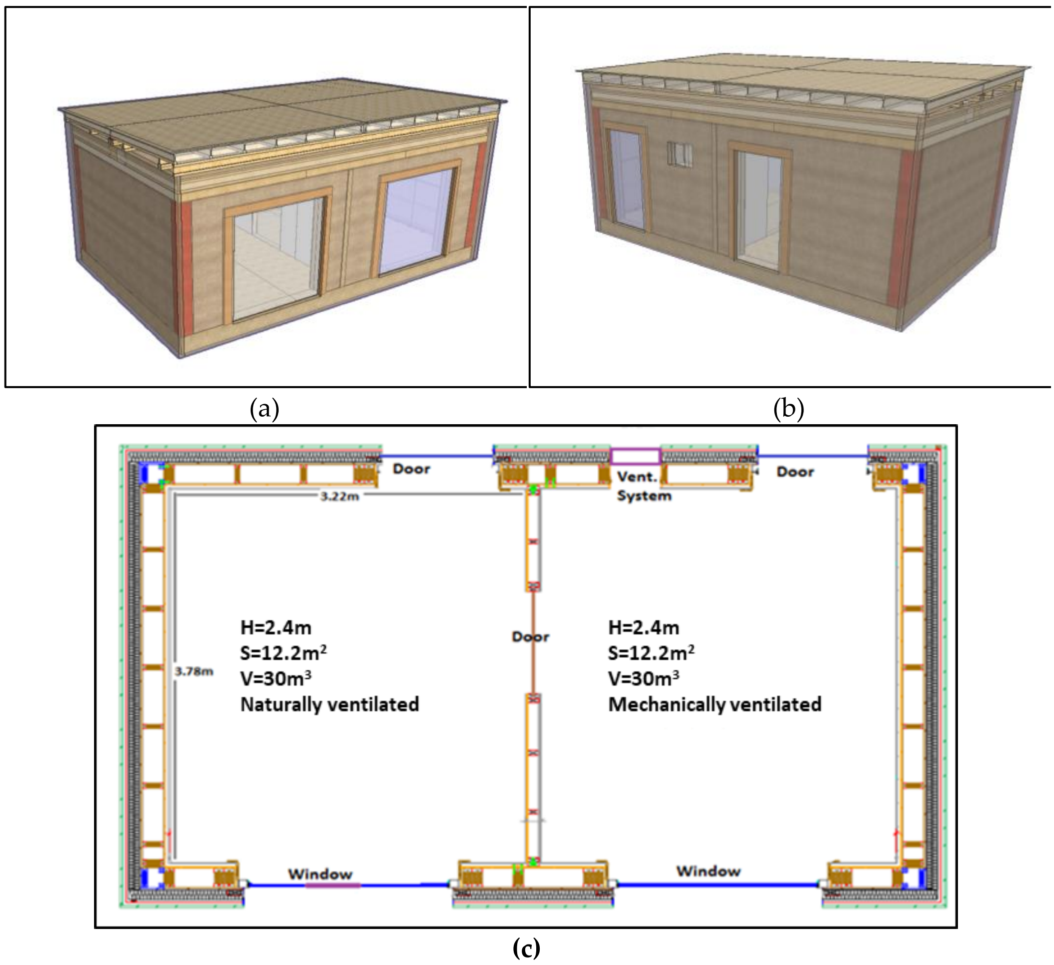

Figure S1. Schematic of the measurement facility: (a) front side, (b) back side, (c) floor plan of the instrumentation room (left) and the experimental room (right).

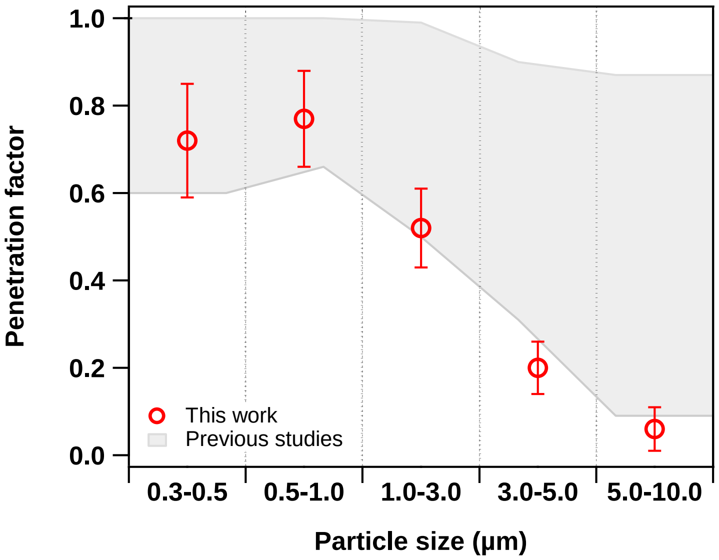

Figure S2. Average values of penetration factors measured during the intensive campaign as a function of particle size. Error bars represent ± 1σ. Grey area represents the range of values observed in previous published studies ([1] and references therein).

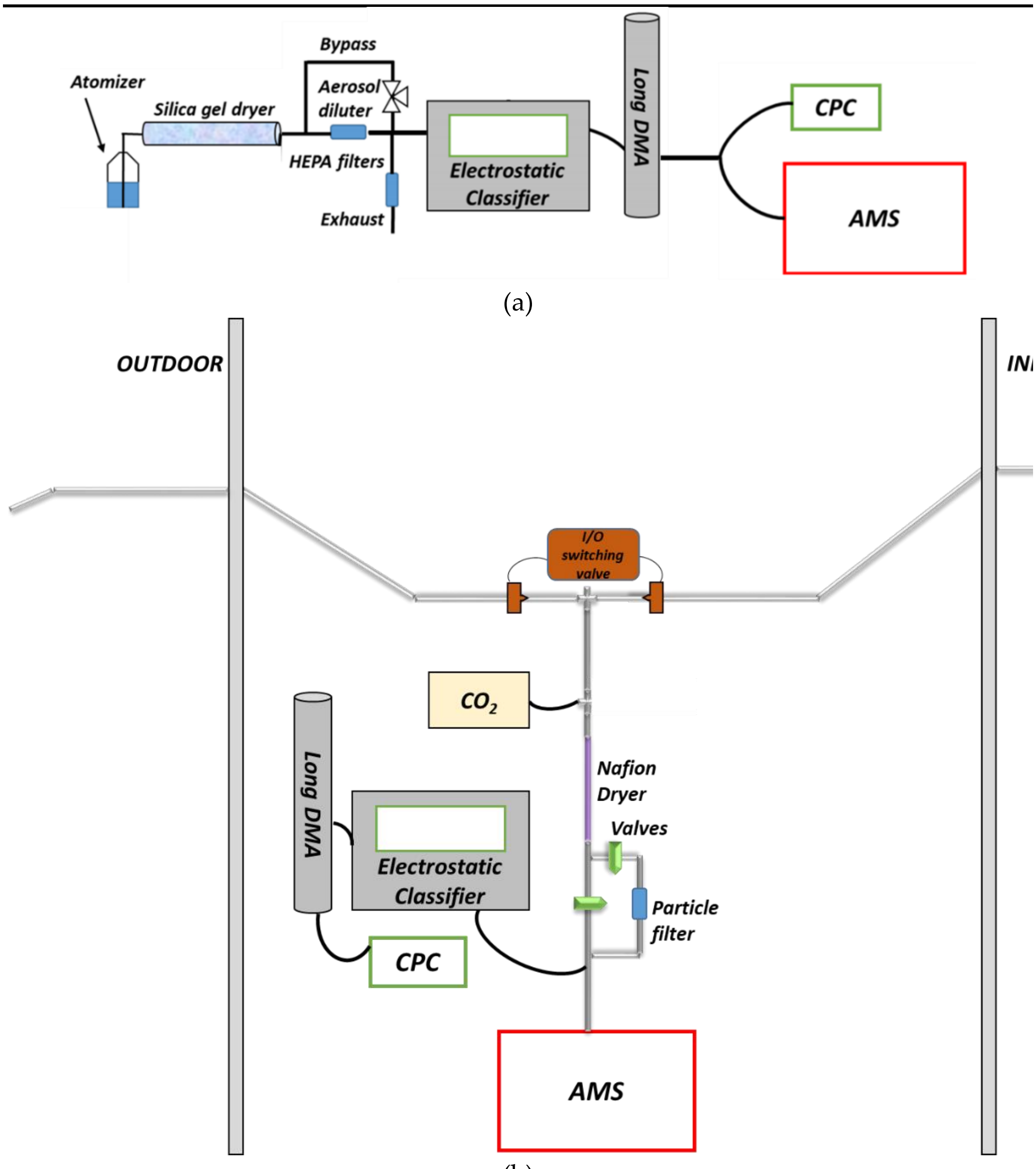

Figure S3. Schematic of (a) AMS calibration unit and (b) AMS sampling configuration with the CPC, SMPS and CO_2 analyzer.

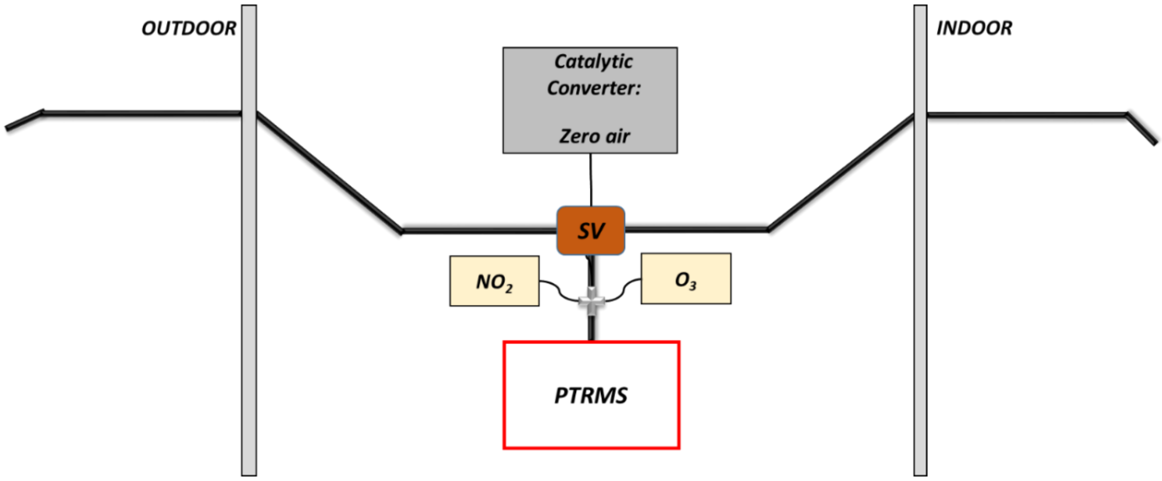

Figure S4. Schematic of the PTRMS setup with the O_3 and NO_2 analyzers.

1. PTRMS measurements

Calibration—The ion transmission curve (transmission vs. m/z) was determined using a gas standard containing the compounds listed in Table S1 covering from m/z 33 to 147. A catalytic converter containing platinum wool (Shimadzu, high sensitivity catalyst for Total Organic Carbon (TOC analyzer) heated to 350 °C was used to generate zero air from ambient air. During calibration, a flow of 1000 sccm of zero air was mixed with 10 sccm of the calibration gas to reach mixing ratios in the range of ~10 ppb. Calibrations were conducted before (n = 6), during (n = 1) and after the end (n = 1) of the intensive campaign.

Table S1. PTRMS gas standard.

Compound (m/z)	Volume Mixing Ratio in the Calibration Mixture (ppb)	Uncertainty (%)
Methanol (33.03349)	990	± 6
Acetaldehyde (45.03349)	950	± 5
Acetone (59.04914)	980	± 5
Benzene (79.054)	990	± 5
Toluene (93.0689)	990	± 5
o-Xylene (107.08553)	1020	± 6
1,2-Dichlorobenzene (146.9768, 148.9739, 150.9709)	1020	± 7

Table S2. Calculations of VOC oxidation rates and comparison to the air exchange rate (a = 0.54 h^{-1}).

Chemical Category	Compound	Molecular Formula	k_{OH} cm³ molecule⁻¹ s⁻¹	k_{O3} cm³ molecule⁻¹ s⁻¹	k_{NO3} cm³ molecule⁻¹ s⁻¹	Total first order oxidation rate (h^{-1})*	Assumption break down
Aromatics	Benzene	C_6H_6	1.20×10^{-12}	1.00×10^{-21}	3.00×10^{-17}	2.16×10^{-3}	No
	Toluene	C_7H_8	5.60×10^{-12}	-	6.60×10^{-17}	1.01×10^{-2}	No
	Ethylbenzene	C_8H_{10}	7.00×10^{-12}	-	-	1.26×10^{-2}	No
Dialkene	Isoprene	C_5H_8	1.00×10^{-10}	1.30×10^{-17}	6.50×10^{-13}	0.26	Yes
Monoterpenes	α-pinene	$C_{10}H_{16}$	5.30×10^{-11}	9.60×10^{-17}	6.20×10^{-12}	0.80	Yes
	β-pinene		7.60×10^{-11}	1.90×10^{-17}	2.50×10^{-12}	0.39	Yes
	Limonene		1.70×10^{-10}	2.20×10^{-16}	1.20×10^{-11}	1.72	Yes
Alcohols	Methanol	CH_3OH	8.80×10^{-13}	-	1.30×10^{-16}	1.60×10^{-3}	No
	Ethanol	C_2H_5O	3.30×10^{-12}	-	4.80×10^{-16}	5.98×10^{-3}	No
Aldehydes	Acetaldehyde	CH_3CHO	1.50×10^{-11}	-	2.70×10^{-15}	2.72×10^{-2}	No
	Benzaldehyde	C_7H_6O	1.30×10^{-11}	-	4.00×10^{-15}	2.37×10^{-2}	No
Ketones	Acetone	C_3H_6O	1.90×10^{-13}	-	1.30×10^{-18}	3.42×10^{-4}	No
	MEK	C_4H_8O	1.05×10^{-12}	-	1.80×10^{-17}	1.89×10^{-3}	No
Carboxylic acids	Formic acid	CH_2O_2	4.50×10^{-13}	-	-	8.10×10^{-4}	No
	Acetic acid	$C_2H_4O_2$	7.50×10^{-13}	-	6.40×10^{-19}	1.35×10^{-3}	No
	Propionic acid	$C_3H_6O_2$	1.20×10^{-12}	-	1.70×10^{-17}	2.16×10^{-3}	No

*Total first order oxidation rate of VOCs (reactions OH, NO_3, and O_3) calculated assuming [OH] = 5 × 105 cm^{-3} (0.025 ppt), [NO_3] = 2.4 × 107 cm^{-3} (1 ppt), [O_3] = 4.8 × 1011 cm^{-3} (20 ppb). kOH, kO_3, kNO_3 are bimolecular rate constants for VOC+OH, VOC+O_3 and VOC+NO_3, respectively.

Table S3. Exact protonated masses detected by PTRMS and non-exhaustive list of VOCs monitored at these m/z.

Formula	Name*	m/z**
$C_4H_8.H^+$	Butene, Butanol frag. (-H_2O)	57.070
$C_5H_6.H^+$	Cyclopentadiene, cyclopentene	67.054
$C_5H_8.H^+$	Isoprene	69.070
$C_5H_{10}.H^+$	Pentene + isomers, Pentanol frag. (-H_2O)	71.086
$C_6H_4.H^+$	-	77.037
$C_6H_6.H^+$	Benzene	79.054
$C_6H_8.H^+$	Monoterpenes frag.	81.070
$C_6H_{10}.H^+$	Cyclohexene, frag. hexenol	83.086
$C_6H_{12}.H^+$	Hexene, Hexanol frag. (-H_2O)	85.102
$C_7H_8.H^+$	Toluene	93.070
$C_7H_{10}.H^+$	Cycloheptadiene	95.086
$C_8H_8.H^+$	Styrene	105.070
$C_8H_{10}.H^+$	C8-aromatics	107.0855
$C_8H_{12}.H^+$	Cyclooctadiene	109.102
$C_8H_{14}.H^+$	Octadiene + frag. alcohol	111.117
$C_8H_{16}.H^+$	Octanol fragment (-H_2O)	113.132
$C_9H_{10}.H^+$	Octene + frag. alcohol	119.086
$C_9H_{12}.H^+$	C9-aromatics	121.101
$C_{10}H_{16}.H^+$	Monoterpenes	137.133
$C_{10}H_{18}.H^+$	-	139.145
$C_{15}H_{24}.H^+$	Sesquiterpenes	205.193
	C_xH_yO	
Formula	*Name*	*m/z*
$CH_3OH.H^+$	Methanol	33.034
$CH_3CHO.H^+$	Acetaldehyde	45.034
$C_2H_5O.H^+$	Ethanol	47.049
$C_3H_4O.H^+$	Acrolein	57.034
$C_3H_6O.H^+$	Acetone	59.049
$C_4H_4O.H^+$	Furane	69.034
$C_4H_6O.H+$	MAC+MVK	71.049
$C_4H_8O.H^+$	MEK	73.065
$C_5H_6O.H^+$	Methyl furan	83.050
$C_5H_8O.H^+$	Cyclopentanone, pentenone	85.065
$C_5H_{10}O.H^+$	Pentanal, MBO	87.080
$C_6H_6O.H^+$	Phenol	95.049
$C_6H_8O.H^+$	Dimethyl furan	97.064
$C_6H_{10}O.H^+$	Hexenal, cyclohexanone	99.080
$C_6H_{12}O.H^+$	Hexanal, hexanone	101.096
$C_7H_4O.H^+$	-	105.032
$C_7H_6O.H^+$	Benzaldehyde	107.049
$C_7H_8O.H^+$	Cresols	109.065
$C_7H_{10}O.H^+$	-	111.077
$C_7H_{12}O.H^+$	-	113.095
$C_8H_{10}O.H^+$	-	123.075
$C_8H_{12}O.H^+$	-	125.0942
$C_8H_{14}O.H^+$	-	127.110

$C_8H_{16}O.H^+$	-	129.126
$C_9H_{10}O.H^+$	-	135.041
$C_9H_{14}O.H^+$	-	139.111
$C_9H_{18}O.H^+$	-	143.141
	$C_xH_yO_2$	
Formula	***Name***	***m/z***
$CH_2O_2.H^+$	Formic Acid	47.013
$C_2H_4O_2.H^+$	Acetic Acid	61.028
$C_2H_6O_2.H^+$	Ethylene glycol	63.032
$C_3H_4O_2.H^+$	Methylglyoxal, propanedial	73.028
$C_3H_6O_2.H^+$	Propionic Acid	75.044
$C_3H_8O_2.H^+$	Propylene glycol	77.058
$C_4H_4O_2.H^+$	Furanone	85.026
$C_4H_6O_2.H^+$	Butanedione, unsaturated carboylic acid	87.043
$C_4H_8O_2.H^+$	Butyric Acid, ethyl acetate	89.059
$C_5H_4O_2.H^+$	Furfural (2-furaldehyde)	97.027
$C_5H_6O_2.H^+$	Furfuranol	99.041
$C_5H_8O_2.H^+$	-	101.060
$C_5H_{10}O_2.H^+$	Pentanoic Acid	103.075
$C_6H_4O_2.H^+$	-	109.028
$C_6H_6O_2.H^+$	Benzenediol	111.042
$C_6H_8O_2.H^+$	-	113.060
$C_6H_{12}O_2.H^+$	Hexanoic Acid	117.092
$C_7H_4O_2.H^+$	-	121.065
$C_7H_6O_2.H^+$	Benzoic Acid	123.043
$C_7H_8O_2.H^+$	-	125.058
$C_7H_{10}O_2.H^+$	-	127.074
$C_7H_{12}O_2.H^+$	-	129.086
$C_7H_{14}O_2.H^+$	Heptanoic Acid	131.105
$C_8H_{10}O_2.H^+$	-	139.071
	$C_xH_yO_{\geq 3}$	
Formula	***Name***	***m/z***
$C_2H_4O_3.H^+$	Glycolic Acid	77.021
$C_4H_2O_3.H^+$	-	99.004
$C_4H_4O_3.H^+$	-	101.023
$C_4H_6O_3.H^+$	Oxobutanoic Acid	103.039
$C_5H_4O_3.H^+$	-	113.021
$C_5H_8O_3.H^+$	Oxopentanoic Acid	117.055
$C_4H_6O_4.H^+$	Butanedioic Acid	119.046
$C_6H_8O_3.H^+$	-	129.058
$C_6H_{10}O_3.H^+$	Oxohexanoic Acid	131.068
	Others	
Formula	***Name***	***m/z***
$C_2H_3N.H^+$	Acetonitrile	42.034
mz 133	-	133.098
mz 147	-	147
mz 149	-	149
mz 151	-	151
mz 153	-	153

mz 355.07	-	355.07
[$(CH_3)_2SiO]_5$.H+	Siloxane D5	371.101
mz 373	Siloxane D6	373
mz 429	-	429
mz 503	-	503

MAC: methacrolein; MBO: Methyl Butenol; MEK: Methyl Ethyl Ketone; MVK: Methyl Vinyl Ketone. *Color code: green – selectively monitored, orange – likely selectively monitored, white – species potentially monitored, pink – 50% of values below detection limit in at least one of the environments. **Reported m/z values obtained by the peak fitting procedure.

Table S4. Descriptive statistics of compounds measured by PTRMS.

Compound	Range		Median (avg)		I/O ratio
	IN ($\mu g\ m^{-3}$)	OUT ($\mu g\ m^{-3}$)	IN ($\mu g\ m^{-3}$)	OUT ($\mu g\ m^{-3}$)	
**VOCs ($\mu g\ m^{-3}$)**					
**C_xH_y**					
$C_4H_8.H^+$	1.43–4.05	0.24–3.26	2.10 (2.18)	0.98 (1.08)	2.33
$C_5H_6.H^+$	0.10–0.23	0.01–0.15	0.13 (0.12)	0.04 (0.05)	3.42
$C_5H_8.H^+$	1.13–1.71	0.08–0.75	1.30 (1.35)	0.24 (0.27)	5.85
$C_5H_{10}.H^+$	0.45–0.77	0.04–0.37	0.57 (0.59)	0.12 (0.14)	4.95
$C_6H_4.H^+$	0.01–0.03	0.01–0.03	0.01(0.01)	0.01(0.01)	1.34
$C_6H_6.H^+$	0.65–2.77	0.26–2.66	1.02 (1.13)	0.69 (0.79)	1.54
$C_6H_{10}.H^+$	1.48–2.21	0.03–0.41	1.85 (1.87)	0.16 (0.18)	13.32
$C_6H_{12}.H^+$	0.13–0.29	0.02–0.18	0.17 (0.18)	0.06 (0.06)	3.30
$C_7H_8.H^+$	0.71–4.40	0.16–4.76	1.55 (1.66)	0.99 (1.21)	1.63
$C_8H_8.H^+$	0.06–1.96	0.03–2.34	0.18 (0.33)	0.12 (0.31)	1.40
$C_8H_{10}.H^+$	0.28–2.94	0.11–3.33	0.95 (1.03)	0.80 (0.97)	1.21
$C_8H_{12}.H^+$	0.20–0.31	0.02–0.14	0.24 (0.25)	0.06 (0.06)	4.78
$C_8H_{14}.H^+$	0.25–0.42	0.02–0.21	0.31 (0.32)	0.07 (0.08)	4.65
$C_8H_{16}.H^+$	0.04–0.07	0.005–0.03	0.05 (0.05)	0.01(0.01)	4.88
$C_9H_{10}.H^+$	0.05–0.46	0.01–0.53	0.09 (0.11)	0.07 (0.09)	1.60
$C_9H_{12}.H^+$	0.18–1.99	0.04–2.02	0.50 (0.61)	0.46 (0.56)	1.31
$C_{10}H_{16}.H^+$	4.07–8.02	0.14–2.33	5.47 (5.69)	0.42 (0.53)	13.79
$C_{10}H_{18}.H^+$	0.05–0.10	0.01–0.06	0.07 (0.07)	0.02 (0.02)	4.09
$C_{15}H_{24}.H^+$	0.06–0.14	0.01–0.07	0.08 (0.09)	0.02 (0.02)	4.60
**C_xH_yO**					
$CH_3OH.H^+$	17.3–25.25	0.56–3.67	20.79 (21.27)	1.74 (1.83)	13.59
$CH_3CHO.H^+$	1.92–3.49	0.32–2.18	2.45 (2.46)	0.86 (0.93)	2.91
$C_2H_5O.H^+$	0.25–16.27	0.91–17.99	3.88 (4.95)	4.83 (5.67)	0.79
$C_3H_4O.H^+$	0.33–0.71	0.04–0.45	0.44 (0.46)	0.16 (0.18)	3.19
$C_3H_6O.H^+$	3.41–4.78	0.72–2.30	4.06 (4.07)	1.24 (1.29)	3.26
$C_4H_4O.H^+$	0.30–0.88	0.03–0.68	0.39 (0.44)	0.11 (0.16)	4.01
$C_4H_6O.H^+$	0.11–0.32	0.02–0.27	0.16 (0.18)	0.08 (0.10)	2.16
$C_4H_8O.H^+$	0.39–1.53	0.17–1.51	0.58 (0.61)	0.35 (0.39)	1.66
$C_5H_6O.H^+$	0.02–0.47	0.02–0.58	0.08 (0.11)	0.09 (0.13)	0.83
$C_5H_8O.H^+$	0.15–0.36	0.02–0.27	0.20 (0.22)	0.08 (0.10)	2.75
$C5H_{10}O.H^+$	0.16–0.26	0.04–0.15	0.20 (0.20)	0.07 (0.08)	2.78
$C_6H_6O.H^+$	0.16–0.37	0.02–0.36	0.21 (0.22)	0.07 (0.09)	3.60
$C_6H_8O.H^+$	0.11–0.34	0.01–0.28	0.15 (0.17)	0.06 (0.07)	2.97
$C_6H_{10}O.H^+$	0.17–0.33	0.02–0.20	0.21 (0.22)	0.06 (0.08)	3.18

$C_6H_{12}O.H^+$	0.19–0.35	0.02–0.21	0.24 (0.24)	0.04 (0.05)	5.55
$C_7H_4O.H^+$	0.03–0.15	0.01–0.03	0.12(0.12)	0.01(0.01)	10.94
$C_7H_6O.H^+$	0.56–0.94	0.01–0.08	0.74 (0.76)	0.02 (0.03)	39.05
$C_7H_8O.H^+$	0.06–0.20	0.01–0.24	0.09 (0.10)	0.04 (0.06)	2.53
$C_7H_{10}O.H^+$	0.08–0.20	0.01–0.19	0.10 (0.11)	0.04 (0.05)	2.94
$C_7H_{12}O.H^+$	0.08–0.13	0.03–0.08	0.10 (0.10)	0.03 (0.04)	4.92
$C_8H_{10}O.H^+$	0.02–0.10	0.002–0.14	0.03 (0.04)	0.02 (0.03)	1.51
$C_8H_{12}O.H^+$	0.07–0.15	0.01–0.09	0.09 (0.10)	0.02 (0.03)	3.94
$C_8H_{14}O.H^+$	0.11–0.17	0.01–0.04	0.14 (0.14)	0.03 (0.03)	5.65
$C_8H_{16}O.H^+$	0.11–0.17	0.01–0.04	0.13 (0.13)	0.02 (0.02)	8.00
$C_9H_{10}O.H^+$	0.24–0.77	0.01–0.02	0.44 (0.46)	0.01(0.01)	49.29
$C_9H_{14}O.H^+$	0.12–0.22	0.01–0.08	0.15 (0.16)	0.03 (0.03)	5.66
$C_9H_{18}O.H^+$	0.20–0.29	0.01–0.05	0.24 (0.24)	0.02 (0.03)	9.91
			$C_xH_yO_2$		
$CH_2O_2.H^+$	15.7–29.67	0.60–1.39	21.26 (21.25)	0.93 (0.93)	23.30
$C_2H_4O_2.H^+$	57.4–89.26	0.14–7.54	71.46 (72.69)	3.30 (3.38)	29.63
$C_2H_6O_2.H^+$	0.32–0.51	0.02–0.16	0.40 (0.41)	0.05 (0.06)	7.89
$C_3H_4O_2.H^+$	0.16–0.35	0.03–0.15	0.23 (0.24)	0.07 (0.07)	3.54
$C_3H_6O_2.H^+$	1.42–2.37	0.16–0.86	1.84 (1.84)	0.30 (0.36)	5.88
$C_3H_8O_2.H^+$	0.01–0.04	0.005–0.01	0.03 (0.03)	0.01 (0.01)	4.02
$C_4H_4O_2.H^+$	0.09–0.21	0.02–0.23	0.11 (0.12)	0.06 (0.07)	2.11
$C_4H_6O_2.H^+$	0.43–0.85	0.08–0.56	0.60 (0.59)	0.19 (0.22)	2.96
$C_4H_8O_2.H^+$	0.39–0.88	0.07–0.62	0.52 (0.52)	0.14 (0.17)	3.68
$C_5H_4O_2.H^+$	5.74–9.05	0.10–1.19	7.17 (7.47)	0.40 (0.45)	22.73
$C_5H_6O_2.H^+$	0.10–0.29	0.02–0.32	0.14 (0.15)	0.07 (0.09)	2.05
$C_5H_8O_2.H^+$	0.16–0.36	0.04–0.25	0.21 (0.22)	0.09 (0.10)	2.73
$C_5H_{10}O_2.H^+$	0.16–0.30	0.02–0.07	0.23 (0.23)	0.03 (0.04)	7.07
$C_6H_4O_2.H^+$	0.01–0.03	0.004–0.04	0.01 (0.02)	0.01 (0.02)	1.21
$C_6H_6O_2.H^+$	0.16–0.34	0.01–0.26	0.20 (0.21)	0.05 (0.07)	4.23
$C_6H_8O_2.H^+$	0.06–0.23	0.01–0.43	0.02 (0.23)	0.05 (0.06)	2.03
$C_6H_{12}O_2.H^+$	0.35–0.77	0.03–0.14	0.54 (0.54)	0.06 (0.07)	8.32
$C_7H_6O_2.H^+$	0.18–0.53	0.02–0.15	0.30 (0.31)	0.05 (0.06)	6.30
$C_7H_4O_2.H^+$	0.01–0.40	0.01–0.43	0.08 (0.09)	0.02 (0.04)	5.44
$C_7H_8O_2.H^+$	0.03–0.16	0.01–0.20	0.05 (0.06)	0.03 (0.05)	1.67
$C_7H_{10}O_2.H^+$	0.03–0.10	0.01–0.10	0.05 (0.05)	0.01 (0.03)	2.08
$C_7H_{12}O_2.H^+$	0.07–0.17	0.02–0.15	0.10 (0.11)	0.05 (0.06)	2.04
$C_7H_{14}O_2.H^+$	0.06–0.12	0.01–0.05	0.09 (0.09)	0.02 (0.02)	5.42
$C_8H_{10}O_2.H^+$	0.02–0.11	0.01–0.14	0.04 (0.04)	0.02 (0.03)	1.73
			$C_xH_yO_{z\geq3}$		
$C_2H_4O_3.H^+$	0.01–0.03	0.003–0.01	0.01 (0.02)	0.004 (0.005)	3.54
$C_4H_2O_3.H^+$	0.02–0.07	0.02–0.15	0.03 (0.04)	0.04 (0.05)	0.77
$C_4H_4O_3.H^+$	0.01–0.04	0.01–0.05	0.02 (0.02)	0.02 (0.02)	0.80
$C_4H_6O_3.H^+$	0.29–0.49	0.01–0.08	0.39 (0.39)	0.03 (0.04)	12.63
$C_5H_4O_3.H^+$	0.02–0.06	0.01–0.07	0.03 (0.03)	0.02 (0.03)	1.27
$C_5H_8O_3.H^+$	0.01–0.08	0.01–0.12	0.02 (0.03)	0.03 (0.04)	0.49
$C_4H_6O_4.H+$	0.004–0.05	0.003–0.06	0.01 (0.01)	0.01 (0.01)	1.37
$C_6H_8O_3.H^+$	0.04–0.19	0.01–0.24	0.07 (0.08)	0.07 (0.08)	1.16
$C_6H_{10}O_3.H^+$	0.01–0.04	0.01–0.06	0.01 (0.01)	0.02 (0.02)	0.76
			Others		
$C_2H_3N.H^+$	0.06–0.13	0.04 – 0.13	0.08 (0.08)	0.07 (0.07)	1.10

mz 133.09768	0.10–0.22	0.01–0.15	0.14 (0.15)	0.05 (0.06)	3.12
mz 147	0.04–0.17	0.02–0.20	0.07 (0.08)	0.05 (0.07)	1.37
mz 149	0.09–0.23	0.03–0.20	0.14 (0.15)	0.08 (0.09)	1.91
mz 151	0.24–0.40	0.03–0.21	0.30 (0.30)	0.08 (0.09)	3.75
mz 153	0.18–0.50	0.04–0.56	0.25 (0.26)	0.10 (0.12)	2.56
mz 355	0.02–0.08	0.01–0.08	0.03 (0.04)	0.03 (0.03)	1.36
mz 371	0.02–0.06	0.02–0.06	0.03 (0.03)	0.03 (0.03)	1.22
mz 373	0.02–0.05	0.02–0.05	0.03 (0.03)	0.03 (0.03)	1.13
mz 429	0.02–0.04	< LOD	0.03 (0.03)	< LOD	< LOD
mz 503	0.03–0.06	< LOD	0.04 (0.05)	< LOD	< LOD

Table S1. Descriptive statistics of measured particles (mass and number concentration).

**Compound**	**Range**		**Median (avg)**		**I/O ratio**
	**IN**	**OUT**	**IN**	**OUT**	
**Particles**					
**Number Concentrations (particles m⁻³)**					
SMPS $_{10.2\text{-}414.2\ nm}$	1.60×10^9– 1.23×10^{10}	2.02×10^9– 1.74×10^{10}	5.43×10^9 (5.78×10^9)	7.85×10^9 (8.06×10^9)	0.75
OPC $_{0.3\text{-}0.5\ \mu m}$	4.00×10^6– 6.62×10^7	4.44×10^6– 1.03×10^8	1.21×10^7 (1.72×10^7)	1.66×10^7 (2.59×10^7)	0.70
OPC $_{0.5\text{-}1.0\ \mu m}$	8.61×10^5– 1.01×10^7	1.21×10^6– 1.62×10^7	2.2×10^6 (2.58×10^6)	3.03×10^6 (3.67×10^6)	0.71
OPC $_{1.0\text{-}3.0\ \mu m}$	1.13×10^5– 2.04×10^6	1.96×10^5– 3.76×10^6	4.03×10^5 (4.93×10^5)	7.64×10^5 (9.38×10^5)	0.53
OPC $_{3.0\text{-}5.0\ \mu m}$	$1.17 \cdot 10^3$– 1.19×10^4	3.16×10^3– 1.70×10^5	3.56×10^3 (4.08×10^3)	2.03×10^4 (2.64×10^4)	0.19
OPC $_{5.0\text{-}10.0\ \mu m}$	8.17×10^1– 9.19×10^2	4.11×10^2– 4.84×10^4	2.49×10^2 (3.21×10^2)	2.11×10^3 (3.57×10^3)	0.14
**Mass Concentrations (μg m⁻³)**					
NO_3	0.02–0.39	0.05–6.38	0.44 (0.76)	1.18 (1.72)	0.48
inorgNO_3	0.01–2.75	0.01–5.68	0.21 (0.44)	0.67 (1.17)	0.44
orgNO_3	0.01–0.73	0.05–1.21	0.20 (0.27)	0.97 (0.44)	0.65
SO_4	0.05–1.93	0.07–2.33	0.23 (0.30)	0.27 (0.35)	0.87
NH_4	0.05–1.47	0.04–3.11	0.46 (0.51)	0.71 (1.00)	0.43
Org	0.39–6.20	0.20–9.23	1.59 (2.02)	1.98 (2.56)	0.85
NR-PM_1	0.52–9.56	0.37–14.48	2.75 (3.53)	4.63 (5.65)	0.68
SMPS	1.13–18.09	0.93–24.56	6.87 (7.26)	7.18 (8.89)	0.87
PM_1 TEOM-FDMS	-	0.07–28.1	-	7.4 (8.1)	-
$PM_{2.5}$ TEOM-FDMS ATMO*	-	0.4-29.3	-	9.9 (11.1)	-

*Located 1.5 km away, part of the regional air quality monitoring network

Table S6. Parameters from C_{IN} vs. C_{OUT} scatter plots, indoor (IC) and outdoor contributions (OC), and emission rates (ER) for aerosols and trace gases.

**Parameter**	**Slope**	**σ***	**Intercept (μg m⁻³)**	**RIC**	**ROC**	**ER (μg m⁻³ h⁻¹)**
Particles						
Org[a]	*0.701*	*0.027*	0.225	6%	94%	-
NO_3[a]	*0.456*	*0.014*	−0.024	−85%	100%	-
SO_4	**0.797**	**0.017**	0.017	6%	94%	-

NH_4[a]	0.460	0.033	0.004	−113%	100%	-
$NR\text{-}PM_1$[a]	0.579	0.019	0.260	−15%	100%	-
$SMPS_{10.2–414.2\ nm}$[a]	0.701	0.018	0.152	6%	94%	-
$OPC_{0.3–0.5\ \mu m}$	0.534	0.022	0.130	23%	77%	-
$OPC_{0.5–1.0\ \mu m}$	0.648	0.031	0.056	8%	92%	-
$OPC_{1.0–3.0\ \mu m}$	0.427	0.016	0.387	18%	82%	-
$OPC_{3.0–5.0\ \mu m}$	0.102	0.010	0.640	37%	63%	-
$OPC_{5.0–10.0\ \mu m}$	0.086	0.010	0.021	9%	91%	-
Inorganic Gases						
O_3[b]	0.764	0.029	−0.624	−40%	100%	-
NO_2[b]	0.892	0.031	1.844	15%	85%	-
VOCs						
C_xH_y						
$C_4H_8.H^+$	0.909	0.037	1.200	54%	46%	0.61
$C_5H_6.H^+$	0.963	0.042	0.088	68%	32%	0.05
$C_5H_8.H^+$	0.857	0.104	1.123	83%	17%	0.61
$C_5H_{10}.H^+$	1.021	0.095	0.448	77%	23%	0.24
$C_6H_4.H^+$	1.077	0.101	0.004	62%	37%	0.004
$C_6H_6.H^+$	0.918	0.027	0.403	38%	62%	0.22
$C_6H_{10}.H^+$	0.850	0.298	1.720	92%	8%	0.93
$C_6H_{12}.H^+$	1.076	0.074	0.112	64%	36%	0.06
$C_7H_8.H^+$	0.829	0.034	0.661	44%	56%	0.36
$C_7H_{10}.H^+$	1.242	0.092	0.179	68%	32%	0.10
$C_8H_8.H^+$	0.858	0.030	0.066	32%	68%	0.04
$C_8H_{10}.H^+$	0.805	0.033	0.249	28%	72%	0.13
$C_8H_{12}.H^+$	0.837	0.105	0.199	80%	20%	0.11
$C_8H_{14}.H^+$	0.852	0.083	0.250	79%	21%	0.14
$C_8H_{16}.H^+$	1.008	0.147	0.041	80%	20%	0.02
$C_9H_{10}.H^+$	0.833	0.036	0.039	42%	58%	0.02
$C_9H_{12}.H^+$	0.749	0.042	0.191	36%	64%	0.10
$C_{10}H_{16}.H^+$	1.661	0.183	4.786	84%	16%	2.49
$C_{10}H_{18}.H^+$	1.037	0.076	0.048	71%	29%	0.03
$C_{15}H_{24}.H^+$	1.037	0.170	0.064	75%	25%	0.03
C_xH_yO						
$CH_3OH.H^+$	0.631	0.173	20.040	95%	5%	10.86
$CH_3CHO.H^+$	0.794	0.060	1.724	71%	29%	0.93
$C_2H_5O.H^+$	1.000	0.049	-0.960	-32%	100%	-
$C_3H_4O.H^+$	0.802	0.042	0.318	71%	29%	0.01
$C_3H_6O.H^+$	0.614	0.125	3.149	81%	19%	1.77
$C_4H_4O.H^+$	0.937	0.041	0.291	69%	31%	0.16
$C_4H_6O.H^+$	0.784	0.028	0.100	59%	41%	0.05
$C_4H_8O.H^+$	0.850	0.046	0.275	47%	53%	0.15
$C_5H_6O.H^+$	0.824	0.026	0.0004	-4%	100%	-
$C_5H_8O.H^+$	0.719	0.028	0.147	70%	30%	0.08
$C_5H_{10}O.H^+$	0.691	0.061	0.149	74%	26%	0.08
$C_6H_6O.H^+$	0.544	0.044	0.172	80%	20%	0.09
$C_6H_8O.H^+$	0.837	0.039	0.106	66%	34%	0.06
$C_6H_{10}O.H^+$	0.949	0.107	0.144	67%	33%	0.09
$C_6H_{12}O.H^+$	0.817	0.171	0.201	83%	17%	0.11
$C_7H_4O.H^+$	0.724	0.880	0.116	97%	3%	0.06

$C_7H_6O.H^+$	0.749	1.150	0.755	98%	2%	0.40
$C_7H_8O.H^+$	0.470	0.024	0.069	75%	25%	0.04
$C_7H_{10}O.H^+$	0.696	0.036	0.076	71%	29%	0.04
$C_7H_{12}O.H^+$	0.612	0.027	0.085	86%	14%	0.05
$C_8H_{10}O.H^+$	0.445	0.027	0.024	62%	38%	0.01
$C_8H_{12}O.H^+$	0.819	0.056	0.072	76%	24%	0.04
$C_8H_{14}O.H^+$	1.035	0.0270	0.111	80%	20%	0.06
$C_8H_{16}O.H^+$	0.840	0.312	0.116	89%	11%	0.06
$C_9H_{10}O.H^+$	7.946	6.360	0.368	92%	8%	0.23
$C_9H_{14}O.H^+$	1.794	0.179	0.111	66%	34%	0.06
$C_9H_{18}O.H^+$	0.730	0.397	0.220	92%	8%	0.12
			$C_xH_yO_2$			
$CH_2O_2.H^+$	5.498	1.752	11.324	76%	24%	8.73
$C_2H_4O_2.H^+$	0.556	0.956	67.158	97%	3%	38.45
$C_2H_6O_2.H^+$	0.591	0.132	0.375	91%	9%	0.20
$C_3H_4O_2.H^+$	0.789	0.125	0.177	75%	25%	0.10
$C_3H_6O_2.H^+$	0.670	0.096	1.600	87%	13%	0.87
$C_3H_8O_2.H^+$	2.177	0.378	0.011	50%	50%	0.01
$C_4H_4O_2.H^+$	0.403	0.034	0.092	77%	23%	0.05
$C_4H_6O_2.H^+$	0.631	0.067	0.453	77%	23%	0.59
$C_4H_8O_2.H^+$	0.679	0.068	0.412	79%	21%	0.22
$C_5H_4O_2.H^+$	0.686	0.240	7.231	96%	4%	3.89
$C_5H_6O_2.H^+$	0.621	0.028	0.095	65%	35%	0.05
$C_5H_8O_2.H^+$	0.565	0.087	0.166	76%	24%	0.09
$C_5H_{10}O_2.H^+$	1.180	0.251	0.167	82%	18%	0.10
$C_6H_4O_2.H^+$	0.536	0.027	0.007	50%	50%	0.004
$C_6H_6O_2.H^+$	0.696	0.051	0.162	78%	22%	0.09
$C_6H_8O_2.H^+$	0.702	0.037	0.061	60%	40%	0.03
$C_6H_{12}O_2.H^+$	2.191	0.433	0.384	72%	28%	0.21
$C_7H_4O_2.H^+$	0.721	0.383	0.076	88%	12%	0.04
$C_7H_6O_2.H^+$	1.483	0.376	0.220	74%	26%	0.12
$C_7H_8O_2.H^+$	0.636	0.030	0.030	55%	45%	0.02
$C_7H_{10}O_2.H^+$	0.705	0.037	0.031	62%	38%	0.02
$C_7H_{12}O_2.H^+$	0.616	0.045	0.069	67%	33%	0.04
$C_7H_{14}O_2.H^+$	1.465	0.188	0.061	71%	29%	0.03
$C_8H_{10}O_2.H^+$	0.628	0.040	0.022	57%	43%	0.01
			$C_xH_yO_{z\geq3}$			
$C_2H_4O_3.H^+$	0.765	0.204	0.012	85%	15%	0.01
$C_4H_2O_3.H^+$	0.260	0.033	0.022	62%	38%	0.01
$C_4H_4O_3.H^+$	0.899	0.097	-0.003	-18%	100%	-
$C_4H_6O_3.H^+$	0.844	0.402	0.357	92%	8%	0.19
$C_5H_4O_3.H^+$	0.563	0.047	0.016	53%	47%	0.01
$C_5H_8O_3.H^+$	0.744	0.051	-0.013	-71%	100%	-
$C_4H_6O_4.H^+$	0.787	0.034	0.004	43%	57%	0.002
$C_6H_8O_3.H^+$	0.669	0.024	0.031	39%	61%	0.02
$C_6H_{10}O_3.H^+$	0.607	0.031	0.003	18%	82%	0.001
			Others			
$C_2H_3N.H^+$	0.821	0.027	0.020	25%	75%	0.01
mz 133.09768	0.871	0.058	0.098	68%	32%	0.05
mz 147	0.711	0.024	0.034	45%	55%	0.02

mz 149	0.700	0.038	0.087	60%	40%	0.05
mz 151	0.409	0.123	0.265	88%	12%	0.14
mz 153	0.794	0.097	0.178	66%	34%	0.09
mz 355.070	0.843	0.029	0.013	43%	57%	0.01
mz 371.101	0.860	0.057	0.010	58%	42%	0.01
mz 373	0.995	0.110	-0.003	51%	49%	0.004
mz 429	0.636	0.220	0.024	100%	0%	0.01
mz 503	0.777	0.306	0.042	100%	0%	0.02

[a] Slope of SO_4 has been used for the IC-OC analysis. [b] Slope of C_xH_y has been used for the IC-OC analysis. *Compounds exhibiting a 3σ RSD larger than 50% for the slope were not included in the analysis.

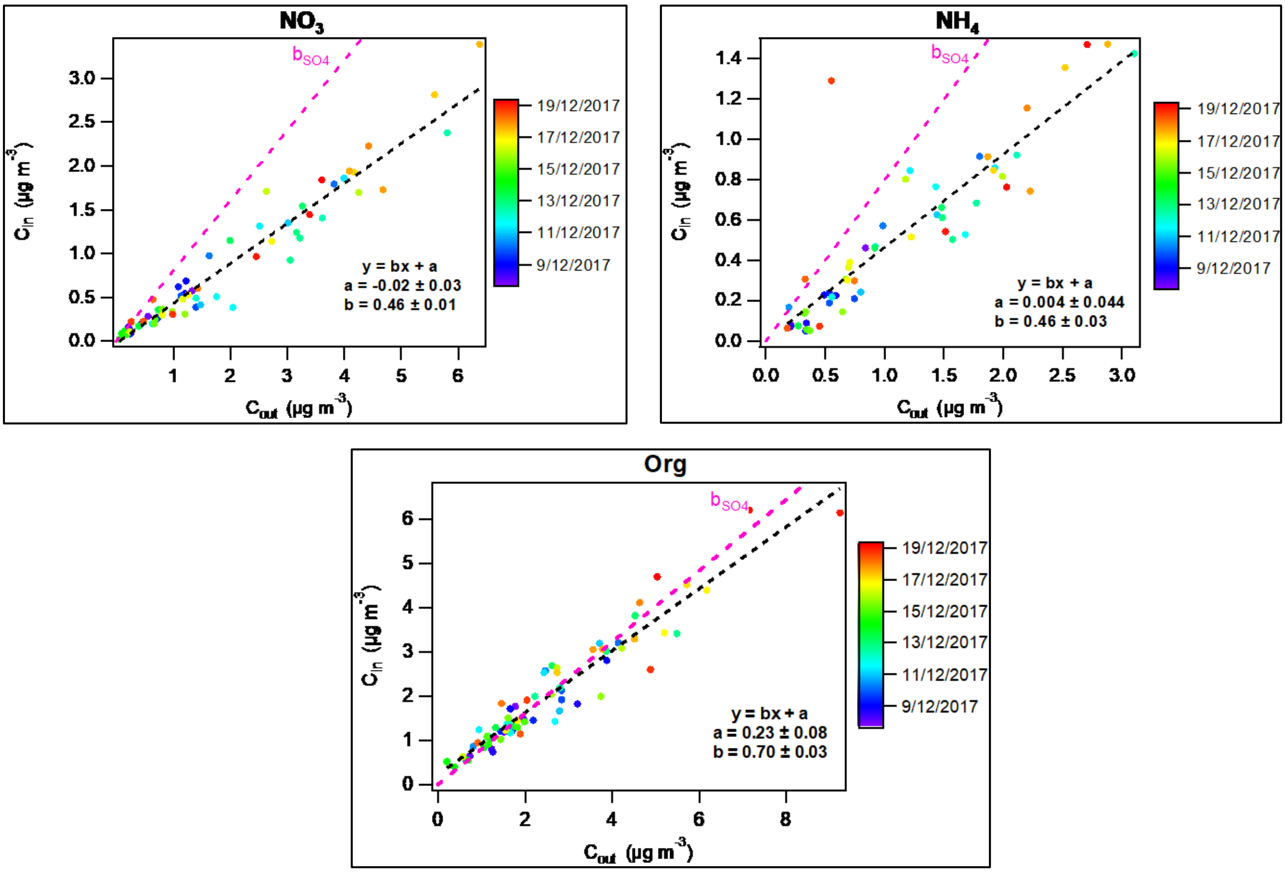

Figure S5. Scatter plots of C_{IN} vs. C_{OUT} for NH_4, NO_3, and Org. Color-coding corresponds to date; dashed black line corresponds to linear fit, dashed pink line corresponds to the slope observed for sulfate.

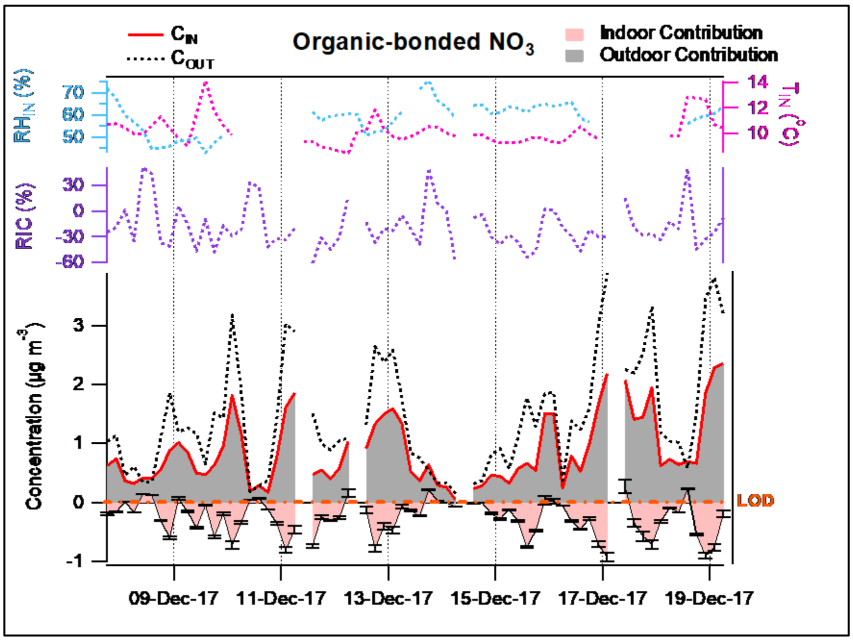

a) IC: -0.27 µg m^{-3}, RIC: -19%

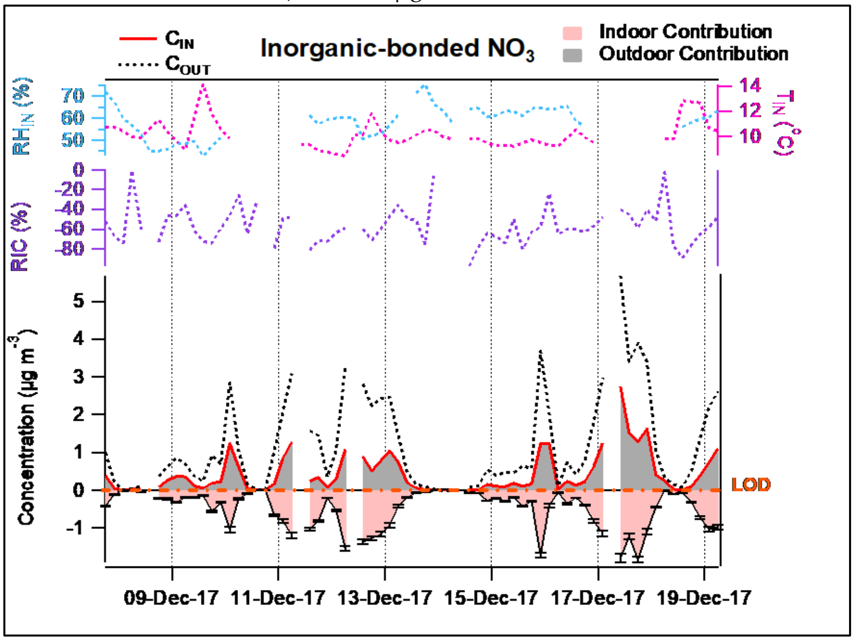

b) IC: -0.50 µg m^{-3}, RIC: -57%

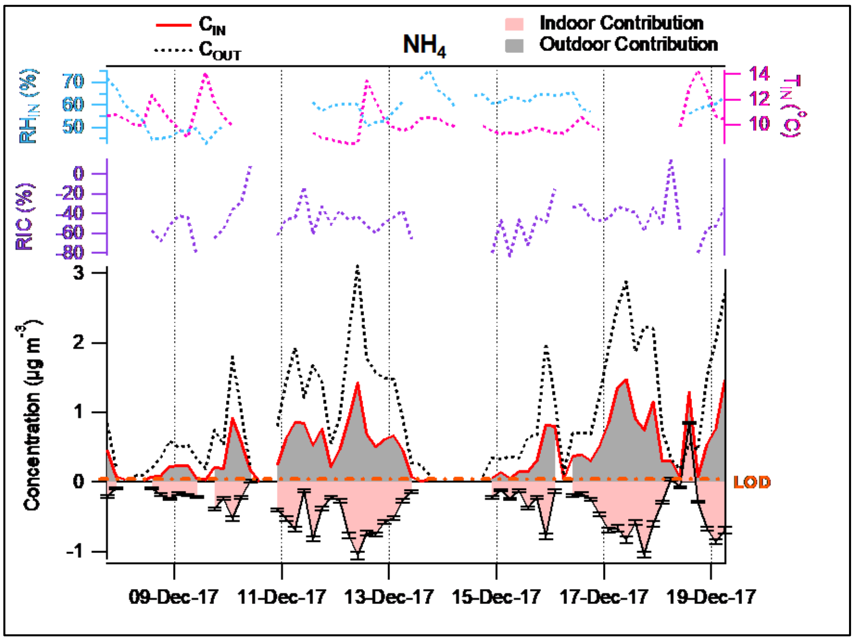

c) IC: -0.38 µg m^{-3}, RIC: -47%

Figure S6. IC-OC contributions (a) orgNO$_3$, (b) inorgNO$_3$ and (c) NH$_4$. Error bars represent 1σ.

References

[1] E. Stratigou, S. Dusanter, J. Brito, and V. Riffault, "Investigation of PM10, PM2.5, PM1 in an unoccupied airflow-controlled room: How reliable to neglect resuspension and assume unreactive particles?," *Building and Environment*, vol. 186, p. 107357, Dec. 2020, doi: 10.1016/j.buildenv.2020.107357.
